# A dry immersion model of microgravity modulates platelet phenotype, miRNA signature, and circulating plasma protein biomarker profile

**DOI:** 10.1038/s41598-021-01335-x

**Published:** 2021-11-09

**Authors:** Laura Twomey, Nastassia Navasiolava, Adrien Robin, Marie-Pierre Bareille, Guillemette Gauquelin-Koch, Arnaud Beck, Françoise Larcher, Gerardene Meade-Murphy, Sinead Sheridan, Patricia B. Maguire, Michael Harrison, Bernard Degryse, Niall M. Moyna, Claude Gharib, Marc-Antoine Custaud, Ronan P. Murphy

**Affiliations:** 1 Technological University Dublin, Dublin 9, Ireland; 2 Cell & Molecular Physiology Group, School of Health & Human Performance, Faculty of Science and Health, Dublin City University, Glasnevin, Dublin 9, Ireland; 3 Univ Angers, CHU Angers, CRC, Inserm, CNRS, MITOVASC, SFR ICAT, 49000 Angers, France; 4 MEDES, Toulouse, France; 5 Centre National d’Études Spatiales (CNES), Paris, France; 6 CHU Angers, Laboratoire de Biochimie, Univ Angers, 49000 Angers, France; 7 Department of Pharmacology and Therapeutics, University College Cork, Cork, Ireland; 8 Department of Sports Science & Physical Education, The Chinese University of Hong Kong, Hong Kong, China; 9 Conway-SPHERE Research Group, Conway Institute, University College Dublin, Dublin, Ireland; 10 Department of Sport and Exercise Science, Waterford Institute of Technology, Cork Road, Waterford, Ireland; 11 Vascular Physiology and Clinical Exercise Medicine Group, School of Health & Human Performance, DCU, Glasnevin, D9 Ireland; 12 Centre for Preventive Medicine, DCU, Glasnevin, D9 Ireland; 13 Institut NeuroMyoGène, Faculté de Médecine Lyon-Est, Université de Lyon, Lyon, France

**Keywords:** Biomarkers, Proteomics, Microarray analysis, Non-coding RNAs, Mechanisms of disease

## Abstract

Ground based research modalities of microgravity have been proposed as innovative methods to investigate the aetiology of chronic age-related conditions such as cardiovascular disease. Dry Immersion (DI), has been effectively used to interrogate the sequelae of physical inactivity (PI) and microgravity on multiple physiological systems. Herein we look at the *causa et effectus* of 3-day DI on platelet phenotype, and correlate with both miRomic and circulating biomarker expression. The miRomic profile of platelets is reflective of phenotype, which itself is sensitive and malleable to the exposome, undergoing responsive transitions in order to fulfil platelets role in thrombosis and haemostasis. Heterogeneous platelet subpopulations circulate at any given time, with varying degrees of sensitivity to activation. Employing a DI model, we investigate the effect of acute PI on platelet function in 12 healthy males. 3-day DI resulted in a significant increase in platelet count, plateletcrit, platelet adhesion, aggregation, and a modest elevation of platelet reactivity index (PRI). We identified 15 protein biomarkers and 22 miRNA whose expression levels were altered after DI. A 3-day DI model of microgravity/physical inactivity induced a prothrombotic platelet phenotype with an unique platelet miRNA signature, increased platelet count and plateletcrit. This correlated with a unique circulating protein biomarker signature. Taken together, these findings highlight platelets as sensitive adaptive sentinels and functional biomarkers of epigenetic drift within the cardiovascular compartment.

## Introduction

Deciphering the aetiology of chronic diseases presents a continual challenge for basic, translational and clinical research. Pertinent to these endeavours is our understanding of cardiovascular disease (CVD) and associated co-morbidities. Efforts in coping with the health burden of CVD requires in depth knowledge of the causative risk factors in order to develop cost-effective preventive, management and treatment strategies. CVD risk factors can track from childhood into adulthood and are strong predictors of subclinical disease in early life^1^. Up to 80% of CVD may be prevented if modifiable risk factors, e.g. physical inactivity are evaded^2^.

The haemostatic system is a complex ancestral pathway which is physiologically adapted to maintain haemostasis and protect vascular integrity. Risk factors perturb homeostasis, contributing to chronic inflammation and CVD in which platelets play a pivotal role. Moreover, platelets have diverse roles and are involved in many inflammatory conditions. Therefore, depending on the physiological context and the environmental architecture, platelet function may be protective or, conversely, contribute to adverse thrombotic and inflammatory outcomes.

Platelets are small, anucleate cells that travel as resting discoid fragments in the circulation. Their average circulating life span is 8–9 days and they are derived from haematopoietic stem cells (HSC) located in the bone marrow niche. Studies have identified the existence of multiple stem cells types within the various bone cellular niches^3,4^. Nevertheless, the dynamics and differentiation steps by which HSCs give rise to the diverse cell types are difficult to characterise due to modelling limitations as well as the complexity of the cellular and molecular processes involved^5,6^.

Platelet biogenesis, is an finely orchestrated series of cellular processes known as *megakaryocytopoiesis* and *thrombopoiesis*. These distinct, non-mutually exclusive processes are driven by temporal, spatial and stochastic patterns of gene expression, thought to be determined by precise epigenetic mechanisms. Epigenetics involves the intricate interplay between genes and the exposome and is fundament to developmental biology. Several recent studies have demonstrated that epigenetic alterations, mostly encompassing DNA methylation, histone tail modifications, and the biogenesis and effector function of microRNAs and other non-coding RNAs (ncRNAs), play integral role in both primary and secondary haemostasis^5,7,8^.

Epigenetic mechanisms display a plasticity in that they are amenable to perturbations throughout our lifespan and drive cell proliferation, differentiation, function and adaptation from conception into adulthood. Environmentally induced epigenetic modifications, such as those elicited by nutrition, stress, pollution, medication and physical inactivity, can influence morbidity and mortality associated with chronic illness and numerous adult onset diseases^9^.

Epigenetic control of haemostasis and thrombosis via mechanisms such as DNA methylation and histone modification is a new domain. Limitations in this area of research have hindered progress, as analysis of these processes requires DNA, which is lacking in platelets^6^. New studies have implicated several key molecular pathways involved in platelet aggregation as being regulated by DNA methylation. Examples of such biochemical arbiters of platelet phenotype and function include the platelet receptors platelet-endothelial aggregation receptor 1 (PEAR1)^10^, protease-activated receptor 4 (PAR4)^11^, glycoprotein VI^12^, and P2Y12^13^. Qualitative and quantitative analysis of discernible platelet phenotypes based on multi ‘omics’ are being associated with a variety of physiological conditions and sensitivity to anti-thrombotic therapeutics^8,14,15^. Various platelet derived parameters known as platelet indices are being employed as a measure of platelet heterogeneity and as surrogate index markers of megakaryocytopoiesis variation^16^. The molecular basis and significance of platelet phenotypic variation remain vague as they do not lend themselves to *lex parsimoniae.*

Scientific studies to elucidate the impact of the exposome on platelet biogenesis are emerging as exciting and active fields of research. Our understanding of the adaptive mechanisms underlying the physiological, cellular and molecular responses to physical inactivity, such as altered platelet phenotype is vague. Our knowledge of physical inactivity is somewhat indirect and is mainly based on the positive effects of exercise training on the sedentary population^17^. As a sedentary lifestyle is often associated with obesity and overweight, some mechanisms involved in the pathogenesis of physical inactivity are similar to that of obesity such as insulin resistance, hypertension, and increased inflammation^18^.

One model being explored to decipher the aetiology of CVD is microgravity (μG). Gravity is a continual force impacting biology and physiology, with living organisms adapting to alterations across the gravitational continuum. Variations in gravitational levels are perceived at the molecular and cellular levels, inducing adaptive responses that influence dynamic physiological functions. Fighting gravity requires daily physical exercise, thus exposure to μG is associated with enhanced inactivity^19^. μG provides a unique model to study the effects of global enhanced physical inactivity and deconditioning imposed on healthy subjects^17,20–27^. Dry immersion reproduces most of the effects of microgravity, inducing rapid and profound deconditioning^20,24^, similar to those observed in spaceflight^26,28^.

To date there have been no in-depth systematic studies on the effect of DI on platelet biogenesis and concomitant phenotype. Herein, we endeavoured to elucidate the impact of physical inactivity and haemoconcentration induced by 3-Day DI on platelet phenotype as well as the cellular and molecular mechanistic alterations implicated in these adaptations.

## Methods

### Subjects

Twelve healthy non-athletic men aged 26 to 39 years (age 32 ± 1.4 yr, weight 75 ± 2 kg, height 178 ± 2 cm, BMI 23.6 ± 0.4 kg/m^2^, maximal oxygen uptake V̇O_2_max 39 ± 1.1 mL/min/kg, mean ± SEM) participated in this study. All participants were non-smokers, had no history of cardiovascular or other chronic diseases, were not taking medication prior to the experiment, and received a comprehensive clinical assessment. All subjects were informed about the experimental procedures and gave their written consent. The experimental protocol conformed to the standards set by the Declaration of Helsinki and was approved by the local Ethic Committee (CPP Sud-Ouest Outre-Mer I, France) and French Health Authorities (*n° ID RCB: 2014-A 00904-43*).

### General DI protocol

The study was conducted at the MEDES space clinic, Toulouse, France. General DI protocol is described in detail previously^29^. Briefly, protocol included three days of ambulatory baseline measurement before immersion (B-3, B-2, B-1), three days (72 h) of dry immersion (DI1, DI2, DI3) and two days of ambulatory recovery (R0, R + 1). The subjects were asked not to exercise during the 8 days of the experiment. Two subjects in two separate baths underwent DI simultaneously. Thermoneutral water temperature (32.5–33.5 °C) was continuously maintained. Light-off period was set at 23:00–07:00. Daily hygiene, weighing and some specific measurements required extraction from the bath. During these short out-of-bath periods, subjects maintained the − 6° head-down position (“strict” protocol). Total out-of-bath supine time for the 72 h of immersion was 4.7 ± 0.16 h. Otherwise, during DI, subjects remained immersed in a supine position for all activities and were continuously observed by video monitoring. Blood pressure (BP), heart rate (HR), and body weight were measured daily at 07:00. Leg echography has been performed daily to ensure the absence of thrombophlebitis. Onset and end of immersion both occurred at 09:00, therefore morning measurements and samplings on DI1 were performed before immersion, and on day R0—still under immersion. Water intake was ad libitum and diet was the same for all participants (standardized to body weight in energy and nutrients). Daily caloric intake was approximately 2820 kcal for baseline and recovery and 2270 kcal for the immersion period. Daily intake for sodium and potassium was approximately 3–4 g. This 3-day DI allowed for several studies on different domains performed by 8 research groups^25,27,29–33^.

### Blood sampling

Antecubital venous blood samples (trisodium citrate tube) were collected in the morning before breakfast, before DI (B-3; *Pre*), under DI on R0 (70 h of DI; *End*) and after DI on R + 1 (22 h following the accomplishment of 3-day DI; *Recovery*).

### Blood count and platelet indices

Immediately after blood sampling, complete blood count and “platelet count” was performed using the Sysmex XN-3000™ Haematology system. Blood count included RBC, WBC, Hb, Hct. Platelet indices included Platelet count (PLT), plateletcrit (PCT), platelet distribution width (PDW), mean platelet volume (MPV), platelet large cell ratio (PLCR) (www.sysmex.co.uk).

### Preparation of platelets

The preparation of Platelet Rich Plasma, Platelet Free Plasma and leukocyte (CD45) depletion of platelets was as previously described^34^. CD45 depletion of platelets for RNA analysis was carried out using EasySep™ magnetic technology (StemCell). A detailed description with adaptions is outline in Supplementary Material.

### Plasma volume estimation

Percent change in plasma volume on R0 and R + 1 vs. B-3 was calculated using Hb and Hct count (Dill and Costill formula): DPV (%) = 100 × [HbB (1- 0.01Hcti)] / [Hbi (1- 0.01HctB)] – 100, where HbB and HctB are baseline Hb and Hct levels, and Hbi and Hcti are Hb and Hct on days R0 and R + 1, respectively.

### Platelet-free plasma isolation

Platelet free plasma (PFP) was isolated for microvesicle work. In order to generate PFP, a double centrifugation method was employed. Blood was drawn using a 21G needle into a sodium citrate vacutainer (0.32% v/v final concentration). The first 3mls of blood was discarded to avoid contamination from cell fragments or tissue factor from venepuncture being collected. The blood sample was mixed by gentle inversions to ensure even distribution of the anticoagulant. Within 15 min of collection, it was centrifuged at 1550xg for 20 min at room temperature (20–22 °C) to pellet the cells. The supernatant PFP containing the microvesicles (MVs) was carefully aspirated leaving a layer of approximately 0.5 cm undisturbed on top of the cells. The collected PFP was centrifuged again at 13,000×*g* for 2 min to remove any contaminating cells or debris. The PFP was then collected, leaving 20% of the sample at the bottom of the tube to be discarded. The PFP was separated in 250 µl aliquots and stored at − 80 °C until further analysis, at which point it was thawed on ice.

### Human protein biomarker assay—Proseek multiplex immunoassay

Proseek biomarker assays were undertaken in collaboration with Olink, Sweden (www.olink.com). Proseek® multiplex CVD II^96x96^ & Proseek® multiplex inflammation I^96x96^ are high-throughput multiplex immunoassays, each enabling analysis of 92 CVD- or inflammation-related protein biomarkers using 1µL of sample and across samples simultaneously. This high level of multiplexing is achieved by proximity extension assay (PEA) technology. A pair of oligonucleotide-labelled antibodies (Proseek probes) specific for each biomarker are allowed to pair-wise bind to each target protein in the sample. When two Proseek probes are in close proximity, a new PCR target sequence is created by a proximity-depended DNA polymerization reaction. This sequence can then be detected by real time PCR and measured. Proseek assays were performed by Olink Bioscience (Upsala, Sweden) to evaluate the expression of two panels of potential CVD and inflammatory biomarkers. Overlap between panels resulted in the total measurement of 152 biomarkers in the PFP samples. Briefly, 1 μl of each sample or negative control was incubated with the conjugated antibodies at 4 °C overnight (day 1). On day 2, the PEA mixture was added and the products were extended and pre-amplified using PCR (ABI 2720 Thermal cycler, Life Technologies). The detection reagent was added to 2.8 µl of the extended and pre-amplified product, mixed and then loaded into the Fluidigm Gene Expression 96 × 96 Dynamic arrays (Fluidigm Corporation) on one side and the Primer plate with specific primers on the other side of the chip. The chip was primed using Fluidigm IFC controller HX and afterwards loaded into a Fluidigm Biomarker system. Detection and sample analysis was performed by high-throughput real-time PCR analysis using the Fluidigm® BioMark™ HD System. This PCR platform enables simultaneous detection of 96 analytes in 96 samples creating 9,216 data points from a single run.

### Proseek multiplex immunoassay: data analysis

Raw data was analysed using Fluidigm PCR software. The Proseek assay generated Cq values for each biomarker and data was normalized using the extension control and a background value. The data used for statistical analysis was expressed on a log2 scale, where a high value corresponded to a high protein expression and vice versa with a low value. The limit of detection (mean negative control plus 3 × standard deviation) was determined for each biomarker for each sample. The data was normalised and analysed using GenEx software (MultiD, Gothenburg, Sweden). All statistical analyses (dynamic principal component analysis and one-way ANOVA) were performed on normalized data.

### miRNA isolation and amplification

All RNA procedures were undertaken using the strictest sterile techniques using appropriate RNase free consumables and reagents. The *mir*Vana® RNA extraction kit was used for isolation and purification of total RNA from platelet samples as per manufacturer’s instructions. Platelets were prepared as previously outlined and pelleted by centrifugation at 2000xg for 12 min at RT. 400 µl of total lysis binding solution was added to the cells at the commencement of the protocol. The total RNA was then eluted into a fresh collection tube by centrifugation at 10,000×*g* for 30 s using 100 µl of elution buffer, which was pre-heated to 95 °C. The RNA was analysed on a NanoDrop® Spectrophotometer and samples stored at − 80 °C.

### Platelet microRNA and profiling

Platelet miRNA profiles from 8 out of the 12 subjects (due to sample limitations) were assessed at the ***Pre-***DI and ***End-***DI time points (B-3 and R0). Total (non-normalized) leukocyte-depleted platelet RNA (ranging from 3.5 to 7.0 ng) was extracted from equal volumes of platelet rich plasma using the *miRVANA* RNA extraction kit. The miRNA profile was determined by RT-qPCR using Applied Biosystems OpenArray® plate technology on the QuantStudio™ 12 K Flex Real-Time System. For a complete miRNA profile, 754 human miRNAs were quantified.

miRNA profiling was carried out using the Applied Biosystems® TaqMan® Low Density Array (TLDA) Human miRNA A (v2.0) and B (v3.0) cards set. For analysis on the TLDA cards, total RNA was firstly extracted from platelets using the Ambion™ *mir*Vana® miRNA isolation kit as per manufacturer’s instructions. Single stranded cDNA was synthesised from total platelet RNA using the Applied Biosystems TaqMan® miRNA Reverse Transcription (RT) Kit. For a full miRNA profile two RT reactions were needed incorporating primers for both pool A and B miRNA panels. The RT reaction had a final volume of 7.5 µl and contained: 3 µl (1–350 ng) total RNA and 4.5 µl of RT master mix. As the total RNA yield from platelets is generally lower than nucleated cells (less than 350 ng), a preamplification step was carried out prior to committing the cDNA to the TaqMan miRNA arrays to uniformly pre-amplify desired cDNA prior to quantification with the TLDA cards. The sample was diluted with 75 µl of 0.1 × TE buffer (pH 8) and used immediately for array analysis or stored for up at − 80 °C for future use.

A 7900HT PCR system was used for initial miRNA profiling and a QuantStudio™ 12 K Flex Real-Time PCR system was used for large scale profiling. DNA polymerase from the TaqMan® Universal PCR Master Mix amplifies the target cDNA using sequence-specific primers and a probe on the TaqMan microRNA array. 100 µl of the master mix was dispensed into each chamber of the array. The card was centrifuged twice for 1 min at 1000 × g to fill each of the 384 wells of the card in an Eppendorf 5810R centrifuge. OpenArray® plate technology on the QuantStudio™ 12 K Flex Real-Time System (Paris, France) was used for large scale miRNA profiling.

### Bioinformatics analysis of platelet microRNA data

Bioinformatics methods were used to extrapolate biological meaning from miRNA that were significantly up or downregulated after the DI. First, microRNA target prediction was performed using online software tools Targetscan (http://www.targetscan.org/vert_71/) and microRNA.org. Following this, involvement of these targets in pathways of interest from the Kyoto Encyclopaedia of Genes and Genomes (KEGG)^35,36^ was analysed using the Database for Annotation, Visualization and Integrated Discovery (DAVID) (http://david.niaid.nih.gov).

### Platelet function assay with impact-R cone and plate analyser

The Impact-R device tests platelet function in anticoagulated whole blood under near physiological conditions. An image analyser measures the adhered platelets and results are expressed as a percentage of the well surface covered (SC%) as an index of platelet adhesion, and average size of the aggregates (AS µm^2^) as an index of aggregation. Blood samples drawn into sodium citrate vacutainers were analysed 1 h post-draw as per manufacturers recommendations. Blood samples were mixed for 1 min at 10 rpm prior to loading on the apparatus. A 130 µl aliquot of the blood sample was applied to the centre of the well followed immediately by the bell housing and cone on top. The selected programme was started (arterial shear rate of 1800 per second for 2 min). Platelet were stained with 500 µl of May Grunwald stain solution for 1 min. Images were then captured at random. Seven images were captured in total. The software analysed the captured images by eliminating the four least readable images and calculating the average of the remaining three. Results were expressed as SC and AS with a visual and graphical result provided for each test.

### VASP/P2Y_12_ phosphorylation assay

Flow cytometry experiments in this thesis were performed on a Accuri C6 (BD Biosciences). To ensure day-to-day sample reproducibility, all cytometers were calibrated daily. To determine the VASP phosphorylation state of whole blood, an adapted standardized flow cytometric assay (BioCytex, France) was employed^37^. The VASP-P analysis was performed within 4 h after blood collection as per manufacturer’s instructions.

### Microvesicle quantification and analysis

The NanoSight NS300 and Syringe Pump were used to quantify microvesicles (exosomes and microparticles) in PFP samples. Nanoparticle tracking analysis technology (NTA) used in this device combines the properties of light scattering and Brownian motion to attain measurements including concentration and size distribution of particles in a liquid suspension (Methods; Supplementary Material).

### Statistical analysis

Results are expressed as mean ± SEM. Statistical comparisons were performed using a variety of tests, depending on the experimental procedure. Main tests included independent t-tests, paired samples t-tests, repeated measures ANOVA and one-way ANOVA/one-way ANCOVA. Pearson product coefficient was used to examine relationships between variables. Statistical significance was set at a level of 0.05. SPSS v19 statistical package was used to analyse results.

## Results

### Effect of dry immersion on physiological and haematological characteristics

**Figure 1:**
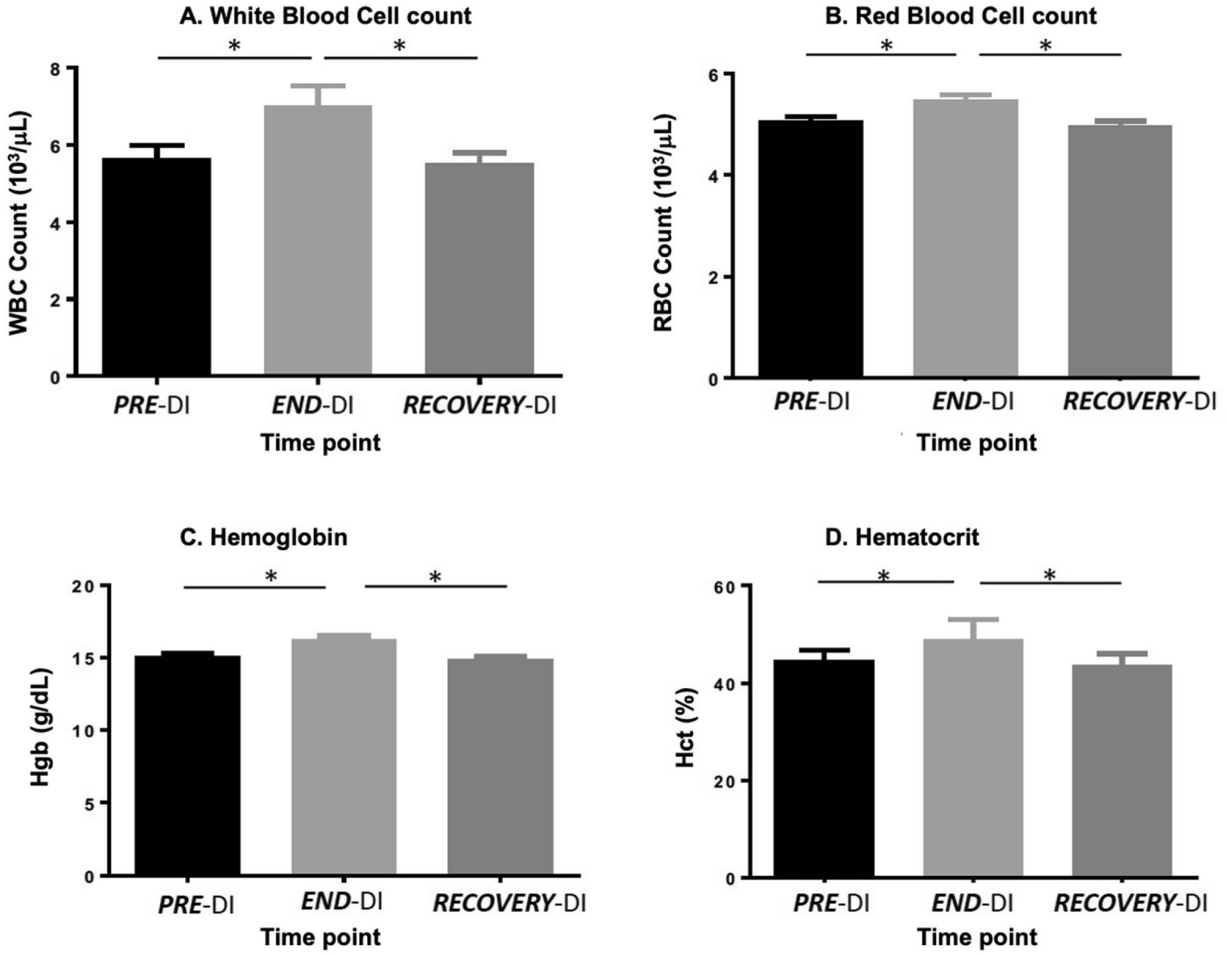
Effect of dry immersion and physical inactivity on RBC and WBC characteristics. Graphs represent the mean ± SEM of each parameter at each time point. (**A**) White Blood Cell count, (**B**) Red Blood Cell count, (**C**) Haemoglobin and (**D**) Haematocrit. *P < 0.05. Paired samples t-test and repeated measures ANOVA (adjusted for age, BMI and VO_2;_ n = 12, biological replicates).

The overview of physiological responses to this 3-day dry immersion protocol has previously been reported^29^. Overall, DI was well tolerated, with no dropouts. Moderate back pain was reported at the beginning, as it is usually seen in dry immersion protocols. HR and BP remained within normal limits throughout the protocol. HR rose slightly at ***END-***DI (57 ± 3 bpm) and ***RECOVERY-***DI (57 ± 2 bpm) vs. ***PRE-***DI (53 ± 2 bpm). SBP and DBP were not significantly modified (Fig. S1; supplementary material). Body weight during immersion decreased by approximately 1–2 kg (Fig. S1). Daily echo-doppler of the lower limbs did not reveal venous problems/thrombosis. Estimated plasma volume showed a 14 ± 2% decrease at ***END-***DI vs. ***PRE-***DI (p < 0.0001). At ***RECOVERY-***DI plasma volume did not significantly differ from ***PRE-***DI (+ 4 ± 2%, p = 0.103). Changes in haematological parameters in response to DI are shown in Fig. 1. There was a significant increase in WBC, RBC concentration, HGB and HCT at the end time point. There was a significant decrease in WBC, RBC concentration and HCT between post and recovery, almost to basal levels.

### Effect of dry immersion on platelet indices

**Figure 2:**
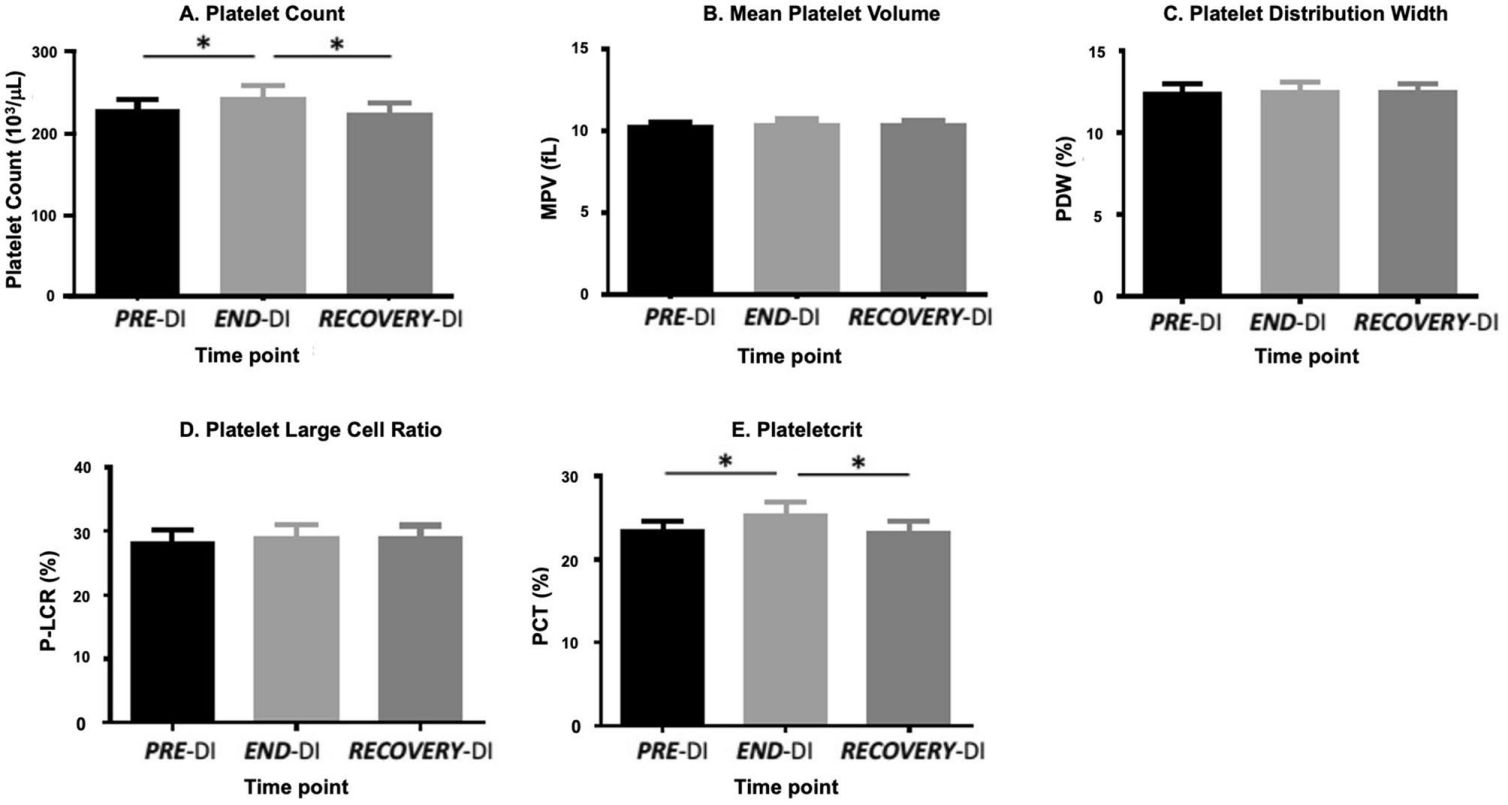
Effect of dry immersion and physical inactivity on platelet indices. Graphs represent the mean ± SEM of each parameter at each time point. (**A**) Platelet count, (**B**) Mean Platelet Volume, (**C**) Platelet Distribution Width, (**D**) Platelet Large Cell Ratio and (**E**) Plateletcrit. * P < 0.05. Paired samples t-test and repeated measures ANOVA (adjusted for age, BMI and VO_2;_ n = 12, biological replicates).

There were no changes in platelet large cell ratio, Mean Platelet Volume and Platelet Distribution Width between any time points. There was a significant increase in platelet count and plateletcrit from ***PRE-***DI to ***END-***DI and subsequently, a significant decrease in these parameters from ***END-***DI time point to ***RECOVERY-***DI (Fig. 2).

### Effect of dry immersion on platelet function

**Figure 3:**
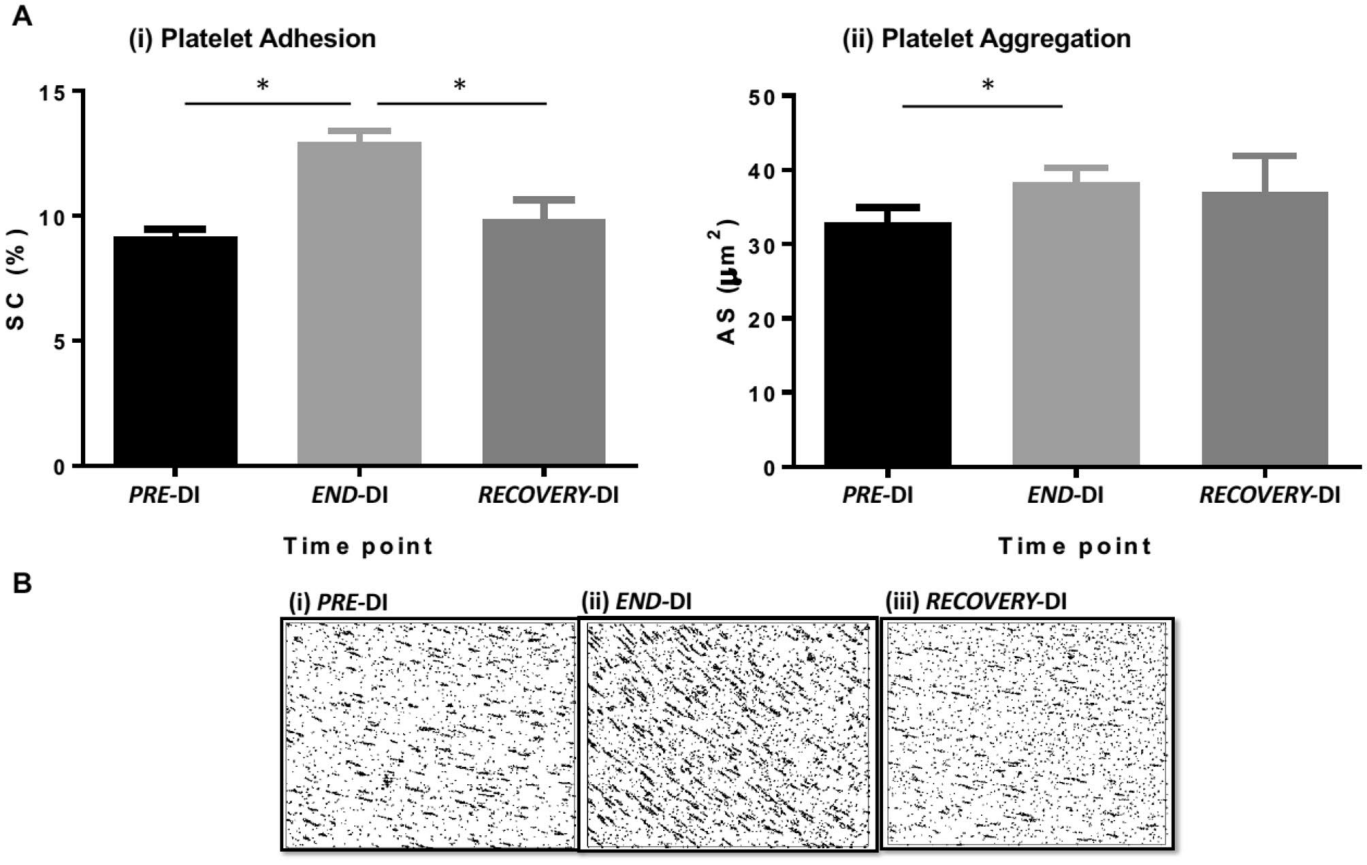
(**A**) Effect of dry immersion on platelet function assessed by Impact R analysis. Graphs represent the mean ± SEM of each parameter at each time point. (i) Platelet Adhesion and (ii) Platelet Aggregation. * P < 0.05. Paired samples t-test and repeated measures ANOVA (adjusted for age, BMI and VO_2;_ n = 12 biological replicates, technical replicates n = 3 for each subject). (**B**) Impact R images from subject J at each stage of dry immersion. Image shows platelet adhesion and aggregation at (i) *PRE-*DI, (ii) *END-*DI and (iii) *RECOVERY-*DI.

Figure 3A,B (representative images from Impact-R (i) ***PRE-***DI, (ii) ***END-***DI and (iii) ***RECOVERY***-DI show the effect of physical inactivity on platelet function. There was a significant increase in platelet adhesion from ***PRE***-DI to ***END***-DI time points and a subsequent decrease between the ***END***-DI and ***RECOVERY***-DI time points. There was also a significant increase in platelet aggregation from ***PRE-***DI to ***END-***DI.

### Effect of dry immersion on platelet VASP phosphorylation

To assess if basal platelet VASP phosphorylation was affected by physical inactivity, the standardised P2Y_12_/VASP kit was used in a flow cytometry assay. The platelet reactivity index (PRI) represents changes in VASP phosphorylation. There was a small insignificant increase in PRI post DI (Fig. S2). There were individual fluctuations in VASP phosphorylation during the DI, possibly reflective of inter-individual variability due to genetic architecture.

### Effect of dry immersion on protein biomarker expression

**Figure 4:**
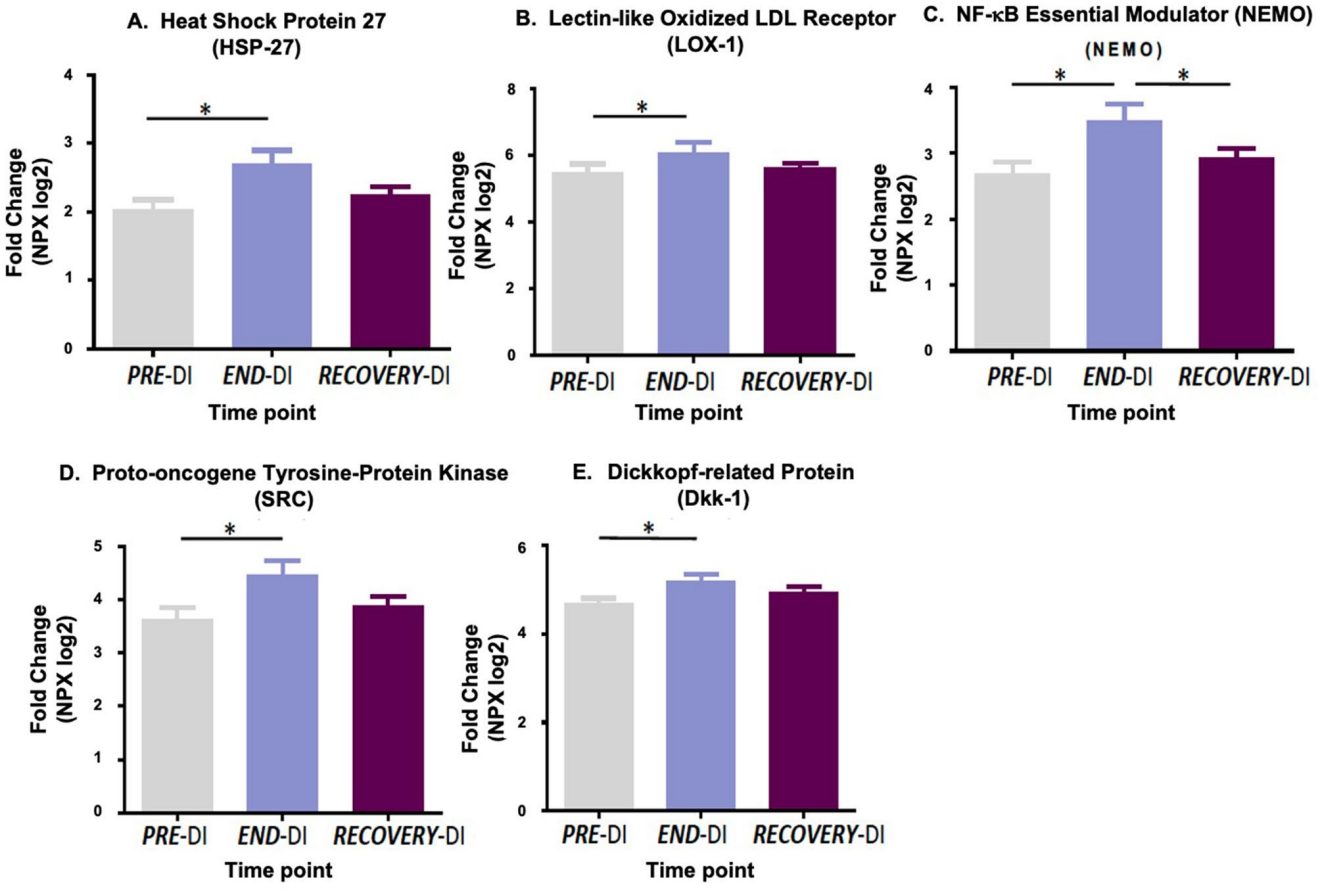
Effect of dry immersion on plasma cardiovascular protein biomarkers. The graphs represent mean ± SEM. (**A**) Heat shock protein 27, (**B**) Lectin like oxidised LDL receptor, (**C**) NF KappaB essential modulator, (**D**) Proto-oncogene tyrosine protein kinase and (**E**) Dickkopf related protein. * P < 0.05, Paired samples t-test and repeated measures ANOVA (adjusted for age, BMI and VO_2_; n = 12 biological replicates).

**Figure 5:**
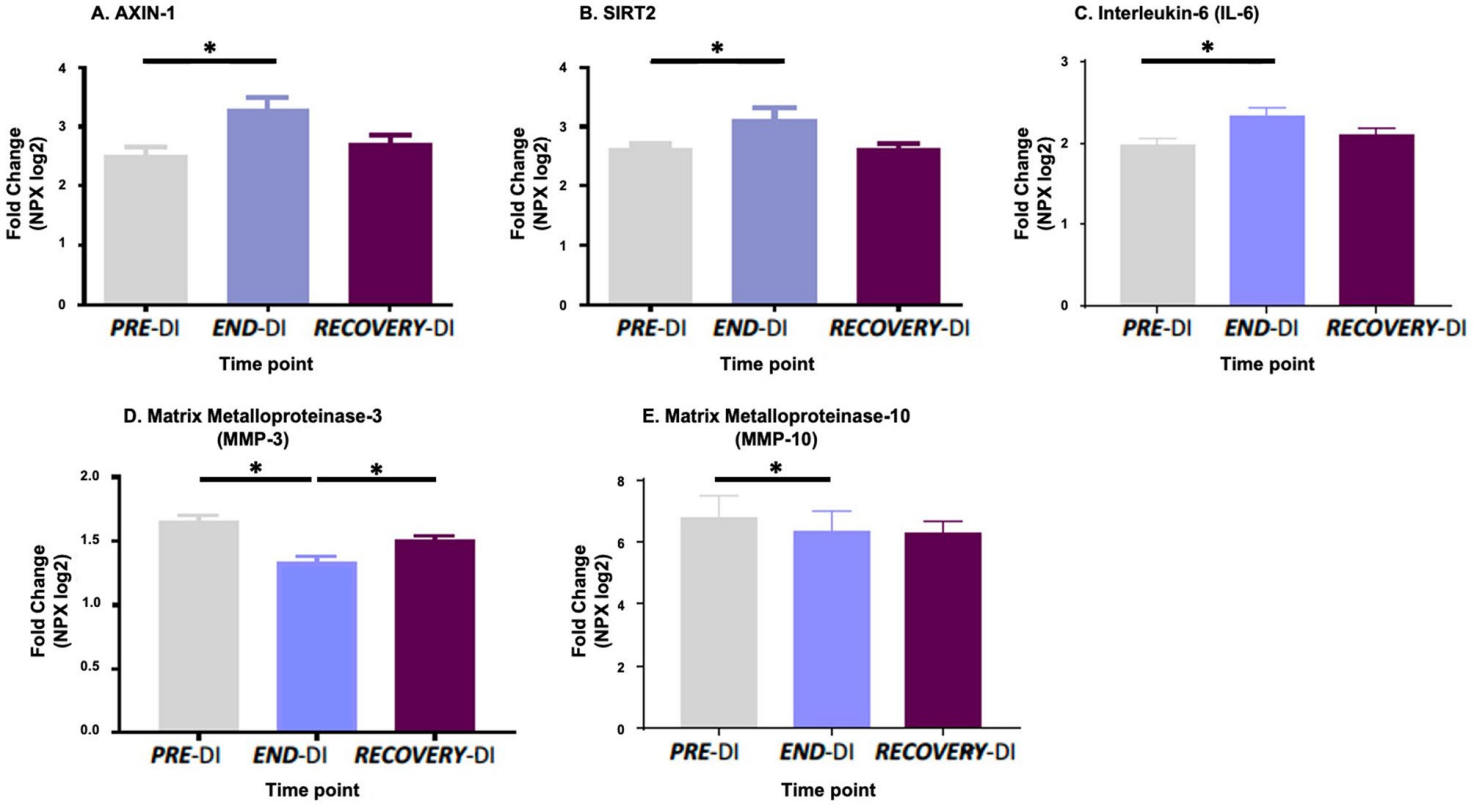
Effect of dry immersion on inflammatory protein biomarkers. All data are expressed mean ± SEM. (**A**) Axin1, (**B**) SIRT2, (**C**) Interleukin-6, (**D**) Matrix metalloproteinase-3 and (**E**) Matrix metalloproteinase 10. Axin1 and MMP-3 were specific to INF panel while Il-6, SIRT2 and MMP-10 were also found on the CVD panel. * P < 0.05, Paired samples t-test and repeated measures ANOVA (adjusted for age, BMI and VO_2_; n = 12 biological replicates).

Protein biomarker expression of platelet poor plasma (PPP) was assessed using the Cardiovascular and Inflammatory protein biomarker panels (Olink Bioscience). The levels of 15 proteins differed significantly between time points (Table 1). Results are expressed as normalised protein expression (NPX) on a log2 scale. Therefore, a normalised increase of 1 is equal to a two-fold increase in protein amount. For 15 proteins out of 131 detected, expression levels differed significantly between different stages of the DI protocol. Seven of these proteins affected by DI are related to platelet function, namely Heat shock protein 27 (HSP27), Lectin-like oxidised LDL receptor (LOX-1), NF-Kappa-B essential modulator (NEMO), Proto-oncogene tyrosine protein kinase (SRC), Dickkopf-related protein (DKK1), Axin1, SIRT2, and IL-6. The changes in biomarker expression levels are displayed in Figs. 4 and 5.

**Table 1:** Protein biomarkers which were differentially expressed after the DI.

Cardiovascular biomarker panel (protein biomarker & main function)	Inflammatory biomarker panel (protein biomarker & main function)
Adrenomedullin (ADM) *Vasodilation and regulation of hormone secretion*	Axin-1 (AXIN1) *Negative regulator of the WNT signalling pathway*
Dickkopf related protein-1 (DKK1) *WNT signalling pathway inhibitor*	Interleukin-6 (IL6) *Pro-inflammatory cytokine*
Heat shock protein-27 (HSPB1) *Stress resistance, actin organization*	STAM-binding protein (STAMBP) *Cytokine-mediated signalling*
Lectin like oxidised LDL receptor-1 (OLR1) *Binds, internalises, degrades oxidized LDL*	Sulfotransferase 1A1 (SULTA1) *Catalyse the sulphate conjugation of hormones, neurotransmitters*
NF-Kappa B essential modulator (IKBKG) *Inflammation, immune genes*	SIRT2 *NB: Possible role in epigenetic gene silencing*
*Renin (REN)* *Activation of angiotensinogen pathway*	Matrix metalloproteinase-10 (MMP10) *Degradation of extracellular matrix*
Proto-oncogene non receptor tyrosine kinase (SRC) *Regulation of cell growth*	Matrix metalloproteinase -3 (MMP3) *Degradation of extracellular matrix*
Tissue plasminogen activator (PLAT) *Disintegration of blood clots*	

The column on the left displays differentially expressed proteins from the CVD panel and the column on the right displays differentially expressed proteins from the inflammation panel.

### Effect of dry immersion on platelet poor plasma microvesicles

Platelet poor plasma (PPP) samples were analysed by Nanosight technology to determine MV size and concentration at each time point. There was a decrease in the average MV size and an increase in average MV concentration at post DI. There was a significant decrease in MV size standard deviation after the DI (Fig. S3). For further analysis, MVs were divided into three categories: Exosomes, microparticles and larger microparticles with modest increases in each category after DI, however the changes were not statistically different.

### Effect of dry immersion on platelet microRNA expression

miRNA expression profiles were analysed from non-normalized leukocyte-depleted platelet RNA (ranging from 3.5 to 7.0 ng) extracted from equal volumes of platelet rich plasma from 8 of the 12 subjects at the time points ***PRE-***DI and ***END-***DI. Western blots of miRNA regulatory proteins are shown in Fig. S4(A) and the number of miRNA either up or downregulated post-DI on the A and B are shown in Fig. S4(B). Heat maps comprised of the most highly expressed miRNA were then constructed for both the A and B panel of miRNA. This allowed visualisation of miRNA expression profiles between subjects, before the DI had been implemented. This is shown in Fig. S5A,B. A shift in colour from red to blue indicates decreasing expression of that miRNA.

### Identification of miRNA affected by physical inactivity

**Figure 6:**
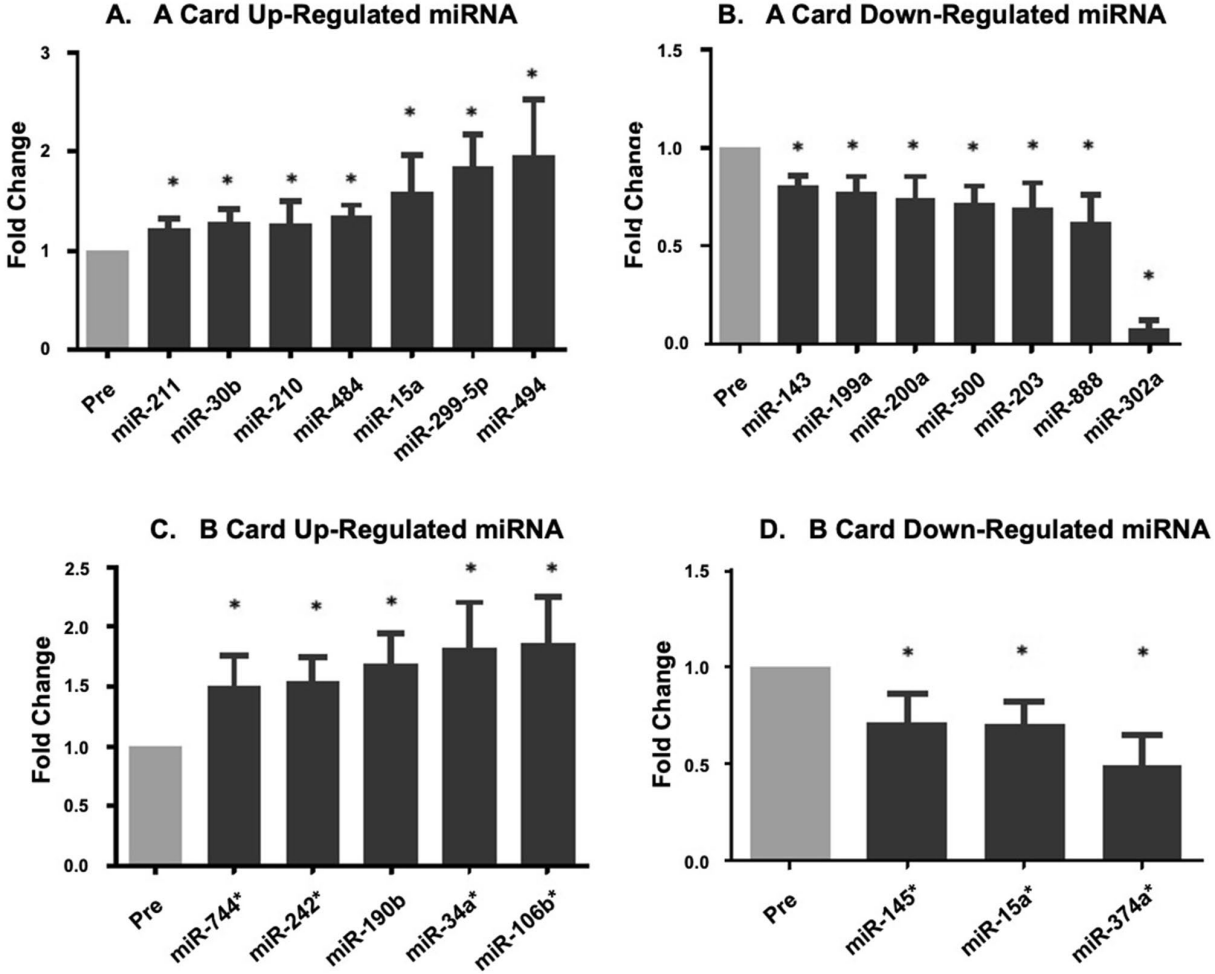
Differentially expressed miRNA at the post dry immersion time point. miRNA profiles were assessed only at the pre-DI and post-DI time points. This figure shows miRNA which were differentially expressed at the post-DI compared to pre-DI time point. All data are expressed mean ± SEM. Graph A shows miRNA that were up regulated after DI on the A card, and graph B shows miRNA that were downregulated after DI on the A card. Graph C shows B card up regulated miRNA, whilst graph D shows B card downregulated miRNA. * P < 0.05, Paired samples t-test and repeated measures ANOVA, n = 8 biological replicates.

**Figure 7:**
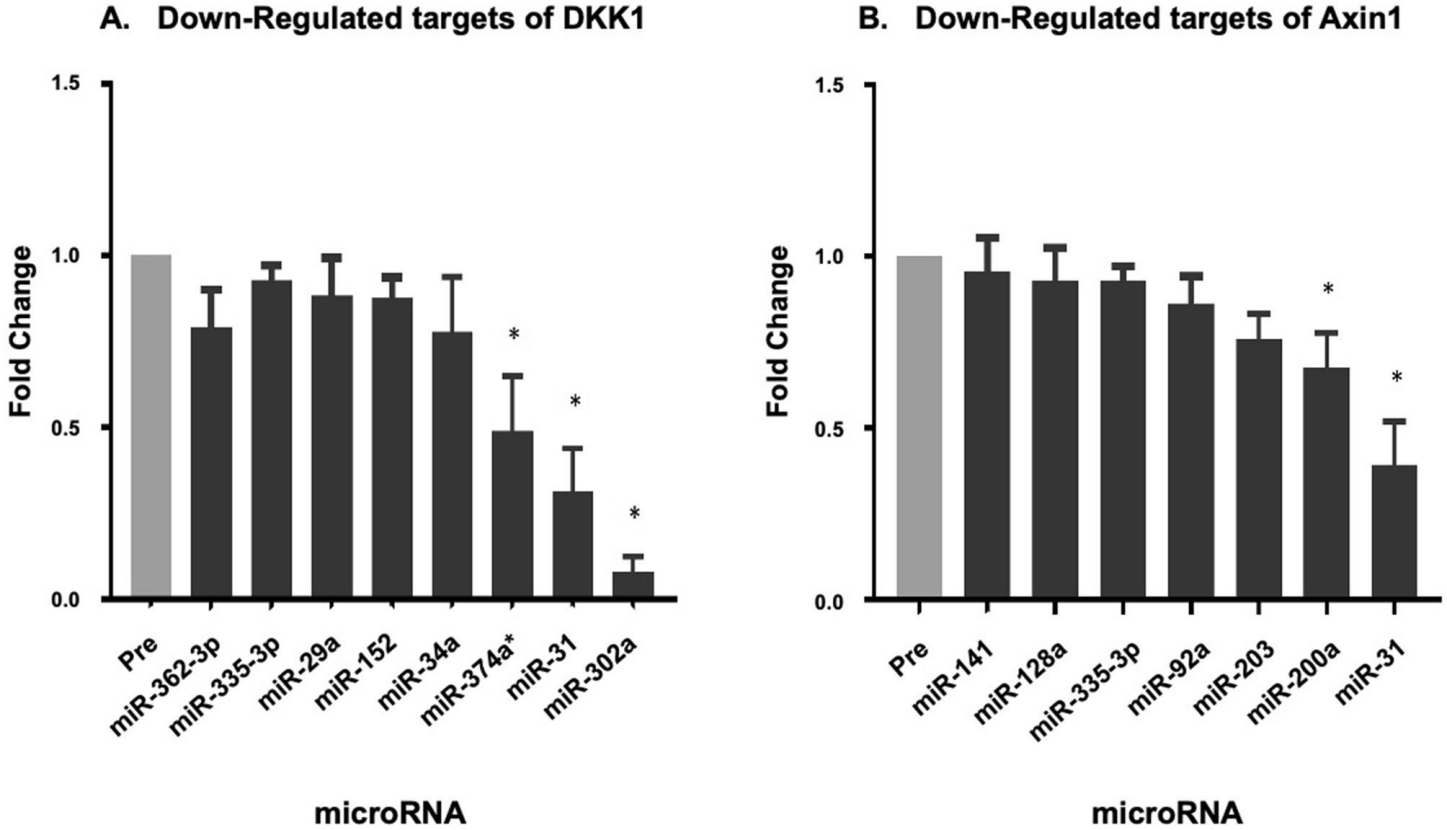
Potential miRNA targeting genes involved in the Wnt signalling pathway. All data are expressed mean ± SEM, n = 8 biological replicates. (**A**) Downregulated miRNA which potentially target DKK1; (**B**) Downregulated miRNA which potentially target Axin1. The differentially expressed miRNA are shown with an asterisk above them, while other potential targets do not have an asterisk.

By comparing the miRNA expression profiles of the subjects between ***PRE-***DI and ***END-***DI, we identified 22 significantly differentially expressed miRNA with a fold change of more than 1.2 (12 upregulated and 10 downregulated). Most reported miRNA fold changes are small (~ 1.5 fold). The miRNAs that were differentially expressed on the A card are shown in Fig. 6A,B, while the miRNA differentially expressed on the B card are shown in Figs. 6C & 7D.

### Bioinformatic analysis of differentially expressed miRNA

To extrapolate biological meaning from miRNA that were significantly up or downregulated after the DI, bioinformatics was performed. This involved determination of putative targets using online software tools including Targetscan and microRNA.org. Following this, involvement of these targets in pathways of interest from the Kyoto Encyclopaedia of Genes and Genomes (KEGG) were analysed using the DAVID bioinformatics database. To help visualise the regulatory potential of each miRNA we examined, a table was constructed illustrating the number of potential genes the miRNA could target and the number of KEGG pathways the predicted targets were part of (Table S2).

### KEGG pathway analysis

Maps of key pathways involved in platelet function and activation were downloaded from the KEGG database using DAVID. Pathways were chosen based on their involvement in platelet function and signalling, specifically in adhesion and aggregation, but also their inflammatory potential. Included in these were the Wnt signalling pathway, regulation of actin cytoskeleton, ECM interaction and Toll-like receptor pathway, of which the Wnt signalling pathway appeared repeatedly in both the up and downregulated miRNA targets. Genes that were potential targets for multiple miRNAs were circled red, genes that were targets for a single miRNA were circled yellow. Potential miRNA targets of genes involved in the Wnt signalling pathway are shown in Fig. S6, potential miRNA targets of genes involved in the reorganisation of the actin cytoskeleton pathway are shown in Fig. S7 and potential miRNA targets of genes involved in the ECM receptor-interaction pathway are shown in Fig. S8.

### miRNA targets of the Wnt signalling pathway

The Wnt signalling pathway involved the largest number of differentially regulated miRNA post DI, as shown previously in Table 2. The protein biomarkers Axin1 and DKK1 were also differentially expressed ***END-***DI. We used online databases to determine additional miRNA which could target Axin1 and DKK1 (Fig. 7A,B). The combined action of multiple down or upregulated miRNA potentially could have affected the gene and subsequent protein expression of Axin1 and DKK1.

**Table 2:** Involvement of differentially regulated miRNA in KEGG cell pathways.

KEGG pathway						
Wnt signalling			Regulation of Actin cytoskeleton		ECM receptor interaction	Toll like receptor
miR-888	miR-500	miR-145	miR-143	miR-34a*	miR-143	miR-143
miR-299a-5p	miR-203	miR-106b*	miR-199a	miR-744	miR-302a	miR-15a
miR-484	miR-302a	miR-34a*	miR-500	miR-494	miR-199a	miR-34a*
miR-199a	miR-190b	miR-24–2	miR-302a	miR-15a*	miR-484	
miR-200a	miR-15a*		miR-374a	miR-106b*		

In silico bioinformatic analysis of differentially regulated miRNA**.** Table shows KEGG pathways common to a number of differentially expressed miRNA and which are involved in platelet function. Permission was kindly granted by the Kanehisa Laboratory for the use of KEGG software.

## Discussion

Despite the strong links between physical inactivity (PI) and CVD risk^1^, there remains a knowledge gap into the effects of PI in healthy subjects. This study allowed a unique opportunity to investigate the effects of acute PI, due to a ground based model of microgravity, on platelet phenotype. Exposure to μG induces a constellation of adaptive physiological modifications, and in particular to the cardiovascular system. DI is characterised by enforced physical inactivity^38^. During DI, the signs of muscle disuse, bone remodelling and spine changes, extensive cardiovascular deconditioning and other alterations mimic the adaptation observed in astronauts. CV deconditioning is a state whereby the CV system does not react efficiently to challenge, distinguished by a reduced capability for exercise, orthostatic intolerance and tachycardia^24^. Initial reactions to DI occur in the first 12 h and are caused by immediate modifications in body fluid distribution and a removal of support structure^20–23^.

In the 1980’s, Kirichenko et al*.* demonstrated that 7 days of DI induced a significant increase in platelet count and hyper-coagulopathic changes to platelet hemostasis^39,40^. A 2011 study examined the effects of simulated microgravity on the miRNA profile of human lymphoblastic cells using a high aspect ratio vessel to model microgravity in space^41^. More recently, 42 miRNAs from cultured human blood lymphocytes from 12 healthy subjects were differentially expressed in microgravity stimulated cells compared to static cells, with resultant mRNA gene targets involved in inflammatory and apoptotic responses^42^. Malkani et al*.* elucidated the role of circulating microRNAs as both a potential biomarker for health risks associated with spaceflight and a countermeasure to mitigate the damage caused to the body by the space environment^43^.

### Haematological parameters

Both spaceflight and its proxies result in an initial plasma volume decrease (approximately 10–15%) after which it remains stable^22,44^. We noted a 14% decrease in plasma volume post DI, with return to baseline at the recovery time point. Blood viscosity usually increases due to the decrease in plasma volume, with a corresponding decrease in RBC is required to maintain blood viscosity^45^. However, decreases in RBC due to altered erythropoiesis takes time, so haemoconcentration was still present at the end of immersion (***END-***DI), reflected by an increase in RBC. There were also significant increases in Hb and HCT during the immersion, although these values remained within the normal limits for healthy individuals. These findings were as expected, as lower plasma volume would result in increased concentrations of RBCs. In general, real and simulated microgravity results in changes to physical properties of RBCs^20,46^. Both a 7-day DI^47^ and 5-day DI^48^ resulted in an increase in RBCs and altered morphological composition of red blood in healthy males. Navasiolava et al*.* reported a significant increase in RBC, Hb and HCT after seven days of DI, which returned to normal after recovery^23^. Unlike our study, they did not observe a significant difference in WBC. However, Bedendeeva et al*.* noted a 40% increase in leukocytes after DI^49^.

### Platelet indices

The effects of DI on platelet indices were investigated as primary indicators of platelet phenotype, and also as surrogate markers of altered megakaryocytopoiesis (Fig. 2). There was an increase in platelet count (PLT) between ***PRE-***DI to ***END-***DI time points, probably reflecting the loss of plasma volume during the DI. There was a significant decrease in PLT between ***END-***DI and ***RECOVERY-***DI suggesting that platelet count returned to pre-immersion levels once subjects began to resume ambulatory activity. Navasiolava et al*.* found no significant difference in PLT after 7-days DI^22^. Other studies have indicated a significant increase in PLT after 7-day DI^39,40^. There were no changes in MPV, PDW or PLCR after three days of DI. There was a significant increase in plateletcrit from ***PRE-***DI to ***END-***DI and a significant decrease in plateletcrit from ***END-***DI to ***RECOVERY-***DI. Plateletcrit appears to be one of the more sensitive platelet indices markers. Overall, the combined increases in PLT and PCT observed at the time-point ***END-***DI may indicate altered megakaryocytopoiesis.

### Platelet function

The effect of PI on platelet function was quantified using the Impact R cone and plate analyser. We observed a significant increase in platelet adhesion from ***PRE-***DI to ***END-***DI suggesting stronger platelet-surface interactions in response to physical inactivity and DI. There was a significant decrease in SC% from ***END-***DI to ***RECOVERY-***DI indicating platelet adhesion levels had returned to their basal state. There was a significant increase in AS from ***PRE-***DI to ***END-***DI suggesting that PI/DI results in elevated platelet aggregation with increased activation of GPαIIbβ3 and increased affinity for fibrinogen binding. Platelet aggregation decreased slightly from ***END-***DI to ***RECOVERY-***DI. The reduction in blood volume as a consequence of reduced total body water in the body during microgravity has been suggested as a factor for thrombotic tendencies, which could have been a determinant of increased platelet activation in this study^50^.

PI causes endothelial dysfunction as well as increasing soluble von Willebrand factor (vWF), tissue factor (TF) expression and endothelial microparticle production, facilitating platelet activation under disturbed flow, inferring that altered shear stress may not activate platelets directly^51^. Haemoconcentration and body fluid redistribution experienced during DI may increase the risk of thrombosis and could also have contributed to enhanced platelet adhesion and aggregation observed after the DI^20,23^. The increase in blood viscosity after 24 h of DI may have been a contributory factor to the platelet hyperaggregability^52^. The use of a viscometer and in parallel with platelet function tests could provide informative data^53^. Interestingly, Kuzichkin et al*.* observed an increase in plasma fibrinogen concentration after short-term space flights and 7-day DI^54^. Assessment of fibrinogen levels and αIIbβ3 activation levels would provide an insight into the increased platelet aggregation ***END-***DI^55^.

### Platelet VASP phosphorylation

VASP is an intracellular regulator of actin dynamics in platelets and plays a key role in regulating platelet adhesion and aggregation^56^. VASP is phosphorylated by cAMP- and cGMP-regulated protein kinases and reflects with inhibition of platelet activation, inhibition of αIIbβ3 and a restriction of VASP to bind to F-actin^57^. Consequently, decreased VASP phosphorylation can result in platelet hyperreactivity. We observed a minor non-significant increase in the average PRI (5%) at ***END-***DI suggesting a reduction in VASP phosphorylation and increase in platelet activation (Fig. S2). Assinger et al*.* showed that VASP phosphorylation at basal levels on the Ser239 residue was significantly reduced in smokers^58^. In response to picomolar and nanomolar concentrations of PGE_1_, smokers still had reduced VASP phosphorylation, which was linked with elevated P-selectin expression. However, at maximal PGE_1_ concentrations they noted no difference between smokers and non-smokers. Similarly, using the VASP/P2Y_12_ kit, PRI (which uses maximal effective doses of PGE_1_) was virtually identical between the smokers and the non-smokers, suggesting that the assessment of VASP phosphorylation in the presence of submaximal quantities of PGE_1_ could be more beneficial. Physical inactivity could produce similar responses and require the same level of investigation.

### Protein biomarker expression

We also examined the effect of PI on platelet poor plasma (PPP) protein biomarkers. The expression profiles of these proteins were analysed at ***PRE-***DI, ***END-***DI and ***RECOVERY***-DI. 131 out of 157 of the proteins were detected in all samples, with 15 proteins significantly ΔDE between different stages of the DI (Table 1). Key proteins affected by physical inactivity included Heat shock protein 27 (HSP27), Lectin-like oxidised LDL receptor (LOX-1), NF-Kappa-B essential modulator (NEMO), Proto-oncogene tyrosine protein kinase (SRC) and Dickkopf-related protein (DKK1) (Fig. 4).

Numerous environmental and physiological stressors mediate the expression of heat shock proteins (HSPs). HSPs have been identified in atherosclerosis^59^ and post exercise^60^. HSP-27 was significantly elevated ***END-***DI suggesting a stress response to the effects of acute physical inactivity. HSP27 has been proposed as a key player in actin polymerisation during platelet shape change, and phosphorylated HSP27 is released from human platelets upon collagen activation, and is associated with the acceleration of platelet aggregation^61^. Moreover, ADP induces phosphorylation of HSP27 with resultant platelet activation markers PDGF and sCD40L release^62^. Elevated HSP-27 ***END-***DI indicates that it was in response to the physiological stress of DI.

Lectin-like oxidised low-density lipoprotein receptor-1 (LOX-1) is a scavenger receptor and is expressed on numerous cells, including platelets, in an activation dependent manner^63^. LOX-1 was significantly elevated ***END-***DI. As LOX-1 recognises and binds to activated platelets, exposure of LOX-1 on the surface of activated platelets might encourage thrombus formation. Furthermore, inhibition of LOX-1 in platelets was shown to prevent platelet aggregation^64^. LOX-1 is associated with obesity and physical inactivity and could represent a marker of platelet activation in response to sedentary behaviour^65^.

DKK1 was significantly upregulated post DI. Platelets represent a major source of circulating DKK1, which is an antagonist of the Wnt signalling pathway and is released from platelet α-granules upon activation^66^. Plasma DKK1 levels are significantly higher in disease states including T2DM and atherosclerosis^66^. DKK1 can also influence platelet-mediated endothelial cell activation involving the Wnt/β-cat signalling pathway and NF-κB pathways.

A number of inflammatory proteins were also differentially regulated ***END-***DI (Fig. 5). Axin-1 is a key member of the Wnt signalling pathway, acting as a scaffold protein and a negative regulator of the Wnt signalling pathway^67^. As Wnt signalling negatively regulates platelet function and modulates the major platelet receptor GPαIIbβ3, we hypothesised that an increase in Axin-1 levels could also have contributed to platelet adhesion and aggregation levels in this study^68–70^.

IL-6, a pleiotropic inflammatory cytokine, was significantly increased ***END-***DI. IL-6 has been adversely linked with sedentary time in a large study of > 500 participants aged ~ 63 years and at high risk for T2DM^71^ and is elevated after physical inactivity in our study. While platelets do not express IL-6, it can affect platelet activation^72^. IL-6 is also a potent thrombopoietic factor promoting maturation of human megakaryocytes in vitro^73^. A number of inflammatory and CVD proteins were ΔDE after 3-day DI, implicating causative effects on both immune and platelet function. Future proteomic studies would prove insightful.

### Platelet poor plasma microvesicles

We investigated the effect of DI on the number and size of MVs in PPP. Figure S3 summarises the changes in circulating MVs in response to DI. There was no change in average MV size ***END-***DI. We found non-significant increases in overall MV concentration. For separate analysis of MV subpopulations, MVs were divided into three distinct categories; Exosomes (30–100 nm), microparticles (100–255 nm), and large microparticles (> 255 nm). There were non-significant increases in exosome and MP concentrations ***END-***DI. Interestingly, there was a non-significant increase in larger MP concentration ***END-***DI, which could suggest the generation of larger and potentially more procoagulant MPs. Studies have shown that endothelial MP levels increased on the third day of a seven-day DI experiment^22^. It has been suggested that an endothelial dysfunction to NO and deterioration in hemodynamic conditions could contribute to increases in EMPs. The changes in endothelial vasodilatory capacity could also have resulted in platelet hyperreactivity and increased MV levels in our study. Platelet-derived MVs represent the most abundant MV source (70–90%) released into blood circulation, and we would expect to see a larger increase in PMPs produced ***END-***DI. Future studies of MVs are warranted.

### Platelet microRNA (miRNA)

To further understand the significance of DI on the molecular mechanisms which underpin platelet phenotype, we investigated miRNA profiles ***PRE-***DI and ***END-***DI, to determine if the miRNome for platelets was altered by physical inactivity. In brief, miRNA are short (18–24) nucleotide long non-coding RNA molecules. They regulate gene expression by hybridising to the 3’ UTR of mRNA. The existence and functionality miRNA in the anucleate human platelets has been described and constitute 80% of all small RNAs in platelets^74^, expressing relatively high quantities of miRNA compared to their nucleated counterparts^75^. Platelet miRNA levels have been demonstrated to be associated with phenotype^76^.

Platelets expressed a total average of 436 miRNA ***PRE-***DI and 438 ***END-***DI, in line to previous similar published studies^74,75^. We identified 22 miRNA which were significantly up or downregulated at ***END-***DI (Fig. 6) of which 10 miRNA were significantly downregulated and 12 miRNA which were significantly up-regulated. Interestingly, miR-374a family has been demonstrated to regulate Wnt/beta-catenin signalling^77^.

As platelets do not express the miRNA nuclear machinery Drosha and DGCR8, the de novo synthesis of new miRNA in platelets is negligible. Upregulation of miRNA in response to physical inactivity could be derived from the processing of pre-miRNA to mature miRNA or as a reflection of increased levels miRNA in their megakaryocyte precursor. As platelets can release MV-containing miRNA upon activation, downregulated miRNA in this study may reflect this process. These findings suggest that platelet miRNAs correlate platelet activation in vitro and may have great potential as biomarkers.

### Bioinformatics of differentially expressed DI regulated miRNA

The fold change, number of conserved targets, and number of pathways the predicted genes are linked to are included in Table S2 for each ΔDE miRNA. Table 2 shows the miRNA potentially involved in the pathways of interest and whether these specific miRNAs were up or downregulated with DI. Pathways were chosen based on their involvement in platelet function and signalling.

Of major interest from the findings of this study was the predicted and potential involvement of ΔDE miRNA on the Wnt signalling pathway, as both DKK1 and Axin-1 proteins were differentially expressed ***END-***DI, and are involved in the Wnt pathway. The literature has described roles for Wnt-β-catenin^69,70^, and non-canonical Wnt signalling pathways in platelet function^78^. Recombinant Wnt3a ligand was shown to inhibit platelet adhesion, shape change, dense granule secretion and inhibiting activation of αIIbβ3 resulting in decreased platelet adhesion to fibrinogen and subsequently reduced aggregation^69^.

DKK1 was one of the significantly upregulated proteins identified from the biomarker panel ***END-***DI and we therefore sought to identify ΔDE miRNA that could target DKK1 (Fig. 7A). A number of these miRNA were downregulated in our study, again possibly indicating a simultaneous downregulation of multiple miRNA targeting DKK1 may act together increasing DKK1 expression. miR-302a, the most down regulated miRNA, has been shown to target DKK1. The increase in circulating DKK1 could negatively regulate the Wnt signalling pathway and ultimately, contribute to elevated platelet adhesion.

Axin-1, a key mediator of the Wnt/β-cat pathway, was identified as one of the proteins that was upregulated after DI^79^. Interestingly, two downregulated miRNAs, miR-203 and miR-200a were identified as potential regulators of Axin-1. Additionally, a number of other miRNA predicted to target Axin-1 were also downregulated as shown in Fig. 7B. This has led us to hypothesis that the simultaneous downregulation of multiple miRNA targeting Axin-1 may explain the observed increase in Axin-1 and result in reduced Wnt signalling in platelets with elevated platelet adhesion and aggregation.

Evidence of physical activity-specific microRNA signatures have seeded the notion that there must also be physical inactivity specific miRNA profiles^80^. Epigenetic variation could be a potential mechanism allowing for independent or perhaps synergistic effects of physical inactivity on platelet function. Hibler et al*.* recently described indications for epigenetic variation and miRNA expression as a link between physical activity and sedentary lifestyle^81^. We hypothesised that an epigenetic adaptation to DI and by association, physical inactivity, is also present inducing epigenetic changes in megakaryocytopoiesis and altered platelet phenotypes.

## Conclusion

**Figure 8:**
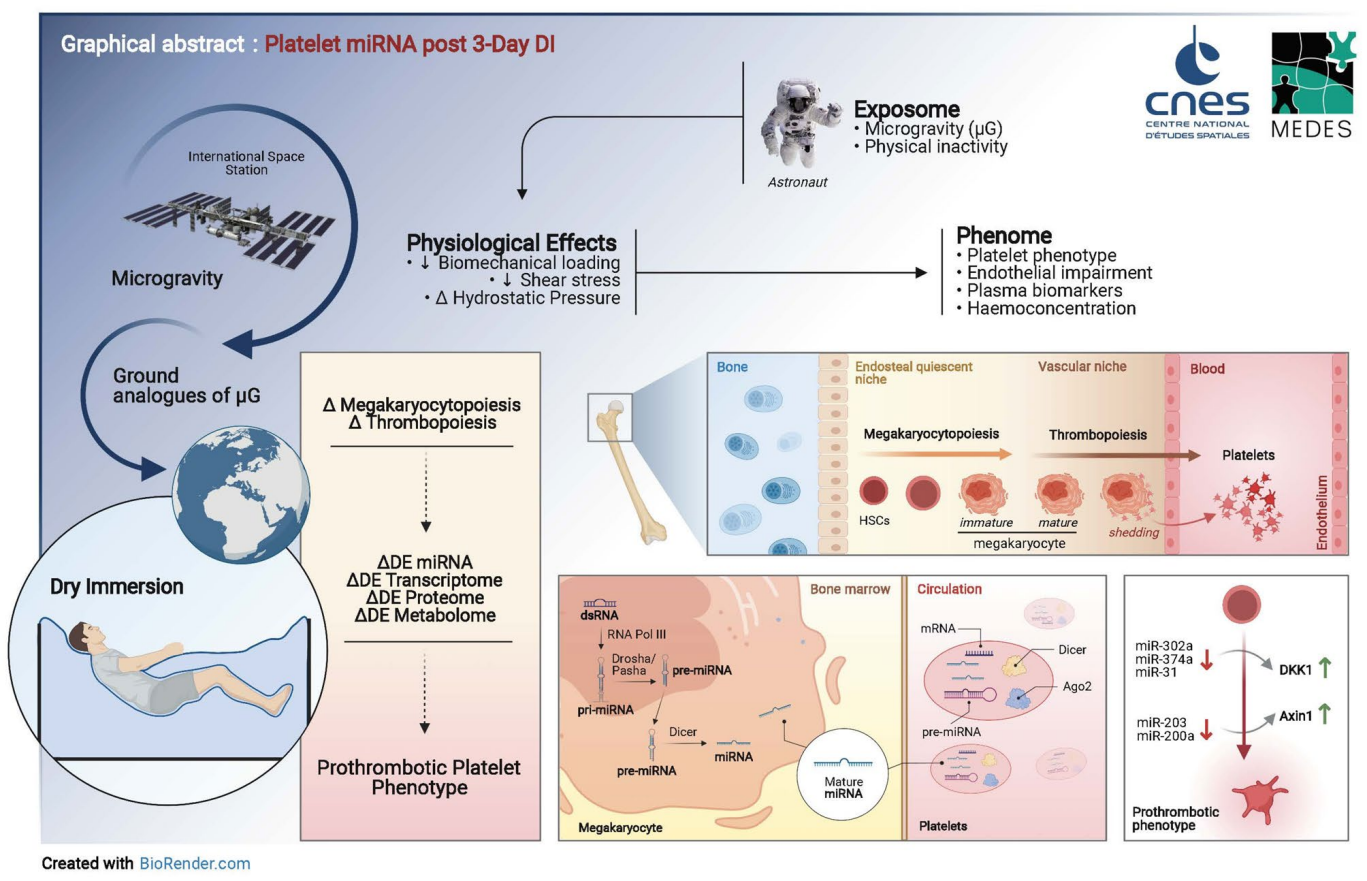
Schematic representation of the findings of this 3-day DI study. Dry immersion, a ground based model of microgravity, alters platelet phenotype to a pro-thrombotic state concurrent with an altered platelet miRNA signature. This finding is also reflected in an altered circulating plasma protein profile for both cardiovascular and inflammatory biomarkers. *Art work by A. Robin & R. Murphy.*

3-day DI induced a rapidly reversible shift to primed platelet phenotype in healthy men, reflected by increased adhesion and aggregation. We have identified 15 ΔDE protein biomarkers associated with 3-day DI. Their expression trends could be of importance for developing ‘biosignatures’ of physical inactivity and CVD risk. We also identified 22 ΔDE platelet miRNA ***END-***DI. These DI related miRNA have potential targets involved in pathways associated with platelet function (Fig. 8). It is evident that the identification of unique signatures of several platelet miRNA, rather than a single miRNA in isolation may enhance diagnostic/prognostic accuracy^82^. The canonical Wnt signalling pathway may signify a novel endogenous mechanism for regulating platelet activity in response to DI. Thus it may provide a countermeasure to mitigate the damage caused to the body by the space environment.

Collectively, our results provide evidence for the early and robust deleterious impact of reduced daily activity on platelet function and phenotype, highlighting the vulnerability of the vasculature to a sedentary lifestyle. It also highlights the importance of physical activity and exercise medicine.

## Supplementary Information

Supplementary Information.

## Abbreviations

ADP: Adenosine diphosphate
ASµm^2^: Aggregate size in µm^2^ units
BMI: Body mass index
cAMP: Adenosine 3’,5’-cyclic monophosphate
CFU-Mk: Colony-forming unit (CFU) assays of megakaryocyte progenitors
cGMP: Guanosine 3,5-cyclic monophosphate
CVD: Cardiovascular disease
DBP: Diastolic blood pressure
DI: Dry immersion
DKK1: Dickkopf-related protein 1
ECM: Extracellular matrix
EMP: Endothelial derived microparticles
Hb: Haemoglobin
HCT: Haematocrit
HR: Heart rate
HSC: Haematopoietic stem cells
HSP27: Heat shock protein 27
IL6: Interleukin-6
KEGG: Kyoto encyclopaedia of genes and genomes
LOX-1: Lectin-like oxidised low-density lipoprotein receptor-1
MFI: Mean fluorescent intensity
miRNA: MicroRNA
MP: Microparticles
MPV: Mean platelet volume
MV: Microvesicles
ncRNA: Noncoding RNA
NEMO: NFKappa-B essential modulator
NPX: Normalised protein expression
NTA: Nanoparticle tracking analysis
PAR4: Protease activated receptor 4
PCT: Plateletcrit
PDW: Platelet distribution width
PEA: Proximity extension assay
PEAR1: Platelet-endothelial aggregation receptor 1
PFP: Platelet free plasma
PGE_1_: Prostaglandin E_1_
PI: Physical inactivity
PLCR: Platelet large cell ratio
PLT: Platelet count
PPP: Platelet poor plasma
pre-miRNA: Preliminary miRNA
pri-miRNA: Primary miRNA
PRI: Platelet Reactivity Index
PRKAR2B: Protein kinase CAMP-dependent type II regulatory subunit beta
PRP: Platelet rich plasma
RBC: Red blood cell count
SBP: Systolic blood pressure
SC%: Surface covered
SIRT2: Sirtuin 2
SRC: Proto-oncogene tyrosine protein kinase
T2DM: Type 2 diabetes mellitus
TAC: Tetrameric antibody complex
TF: Tissue factor
VASP: Vasodilator-stimulated phosphoprotein
VO_2_max: Maximal oxygen uptake
WBC: White blood cell count
ΔDE: Differentially expressed
μG: Microgravity
