# Supplementary material for: A dry immersion model of microgravity modulates platelet phenotype, miRNA signature, and circulating plasma protein biomarker profile
Source: Sci Rep. 2021 Nov 9;11:21906. doi: 10.1038/s41598-021-01335-x (PMC8578674; doi:10.1038/s41598-021-01335-x)
Supplement: Supplementary file 1 — Supplementary Information. [file 41598_2021_1335_MOESM1_ESM.pdf]

Supplementary Material

A dry immersion model of microgravity modulates platelet phenotype, miRNA signature, and circulating plasma protein biomarker profile

Laura Twomey[1,2]*, Nastassia Navasiolava[3]*, Adrien Robin[3], Marie-Pierre Bareille[4], Guillemette Gauquelin-Koch[5], Arnaud Beck[4], Françoise Larcher[6], Gerardene Meade-Murphy[7], Sinead Sheridan[8], Patricia B. Maguire[9], Michael Harrison[10], Bernard Degryse[2,12], Niall M. Moyna[11,12], Claude Gharib[13], Marc-Antoine Custaud[3], Ronan P. Murphy[2,12]✉

[1] Technological University Dublin, Ireland

[2] Cell & Molecular Physiology Group, School of Health & Human Performance, Dublin City University, Glasnevin, Dublin 9, Ireland

[3] Univ Angers, CHU Angers, CRC, Inserm, CNRS, MITOVASC, SFR ICAT, F-49000 Angers, France

[4] MEDES, Toulouse, France

[5] Centre National d'Études Spatiales (CNES), Paris, France

[6] CHU Angers, Laboratoire de Biochimie, Univ Angers, F-49000 Angers, France

[7] Department of Pharmacology and Therapeutics, University College Cork, Cork, Ireland

[8] Department of Sports Science & Physical Education, The Chinese University of Hong Kong

[9] Conway-SPHERE Research Group, Conway Institute, University College Dublin, Dublin, Ireland

[10] Department of Sport and Exercise Science, Waterford Institute of Technology, Cork Road, Waterford, Ireland

[11] Vascular Physiology and Clinical Exercise Medicine Group, School of Health & Human Performance, DCU, Glasnevin, D9, Ireland

[12] Centre for Preventive Medicine, DCU, Glasnevin, D9, Ireland

[13] Institut NeuroMyoGène, Faculté de Médecine Lyon-Est, Université de Lyon, Lyon, France

*Co-authorship, equal contribution

✉ **Corresponding Author:**

Dr Ronan P Murphy *PhD*

School of Health & Human Performance

Faculty of Science and Health

Dublin City University

Glasnevin, Dublin 9

Office: +353-(0)1-700 7357 | Laboratory: +353-(0)1-700 8824

email: ronan.murphy@dcu.ie

	Baseline data collection				Dry Immersion			Recovery	
**Day**	**-x**	**-3**<br>***Pre***	**-2**	**-1**	**1**	**2**	**3**	**0**<br>***END***	**1**<br>***Recovery***
**Volume of Blood for Studies (mls)**		**10**						**10**	**10**

Table S1: Division of blood during the 3-day DI study. Nine millilitres of blood for platelet work were collected on BDC-3 (***PRE-***DI), R+0 (***END-***DI) and R+1 (***Recovery-***DI).

miRNA	Fold change direction	Conserved targets	KEGG Pathways
miR-143	↓	497	24
miR-199a	↓	631	16
miR-200a	↓	896	18
miR-500	↓	185	8
miR-203	↓	964	32
miR-888	↓	3350	36
miR-302a	↓	1022	32
miR-145*	↓	891	22
miR-15*	↓	1508	44
miR-374a*	↓	4289	24
miR-30b	↑	6957	14
miR-211	↑	2809	46
miR-210	↑	4046	38
miR-484	↑	2696	26
miR-15a	↑	3213	33
miR-299-5p	↑	279	1
miR-494	↑	620	28
miR-744*	↑	3696	22
miR-24-2*	↑	741	18
miR-190b	↑	224	10
miR-34a*	↑	4289	22
miR-106B*	↑	698	21

Table S2: Bioinformatics of differentially regulated miRNA. Table of miRNA regulated by DI from the A and B panels. Table shows the number of *in-silico* predicted conserved targets of each miRNA, in addition to the number of GO biological processes and KEGG pathways that these miRNAs are putatively involved in. Permission was kindly granted by the Kanehisa Laboratory for the use of KEGG software.

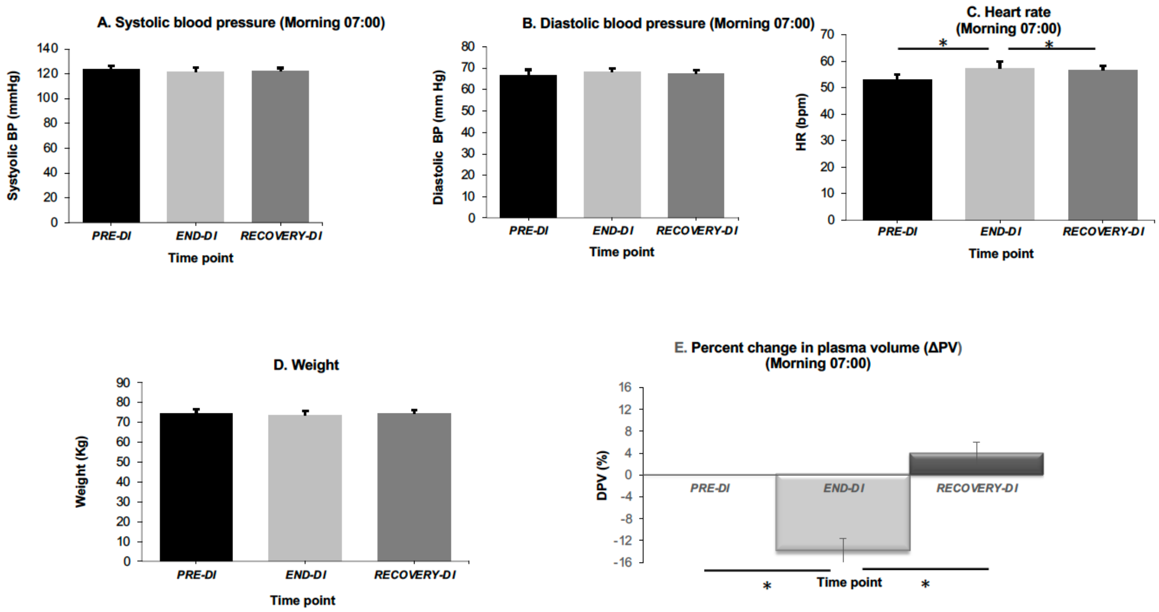

Figure S1: Changes in heart rate, blood pressure, weight and Plasma Volume during the dry immersion. Graphs represent the mean ± SEM of each parameter at each time point. A - Systolic blood pressure, B - Diastolic blood pressure, C - Heart rate, D – Weight, and E - Change in Plasma Volume (ΔPV). * P<0.05, Paired samples t-test and repeated measures ANOVA adjusted for Age, BMI and $VO_{2,}$ biological replicates, n=12).

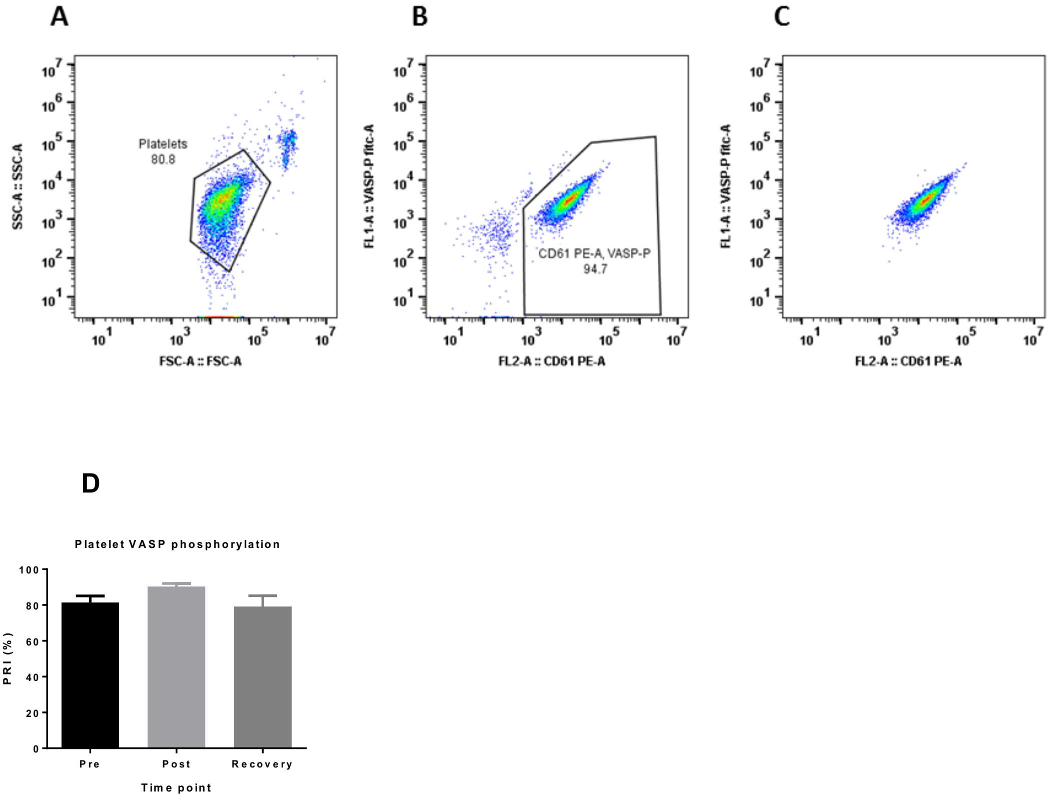

Figure S2: Gating strategy for the analysis of platelet VASP phosphorylation. Platelets were gated by side and forward scatter (A) and then by expression of CD61 (B). VASP-P phosphorylation was quantified by median and geometric mean fluorescence intensities (C). All data was analysed with Flowjo software. **Effect of dry immersion on overall platelet VASP phosphorylation.** Graph represents the mean ± SEM of each parameter at each time point. PRI = Platelet reactivity index. (D). The platelet population was identified by its forward and side scatter distribution and by expression of platelet specific antibody (CD61-PE) and 10,000 platelet events were gated. The value of the "corrected" MFI (MFIc) for each tube was obtained. MFIc was obtained after subtraction of the negative control tube (T3) from the value obtained for VASP-P (tubes one and two). The results from this test are labelled by the manufacturer as the "platelet reactivity index" (PRI) expressed as a percentage change in VASP fluorescence intensity between resting (+PGE_1) and activated (+ADP stimulated) platelets. The PRI % was calculated from the median MFI using the calculation -

PRI % = (MFIPgE1 – MFIPgE1+ADP / MFIPgE1) x 100

For comparisons the PRI was also calculated from the mean fluorescence intensities. The working range of this assay was between 0-100%.

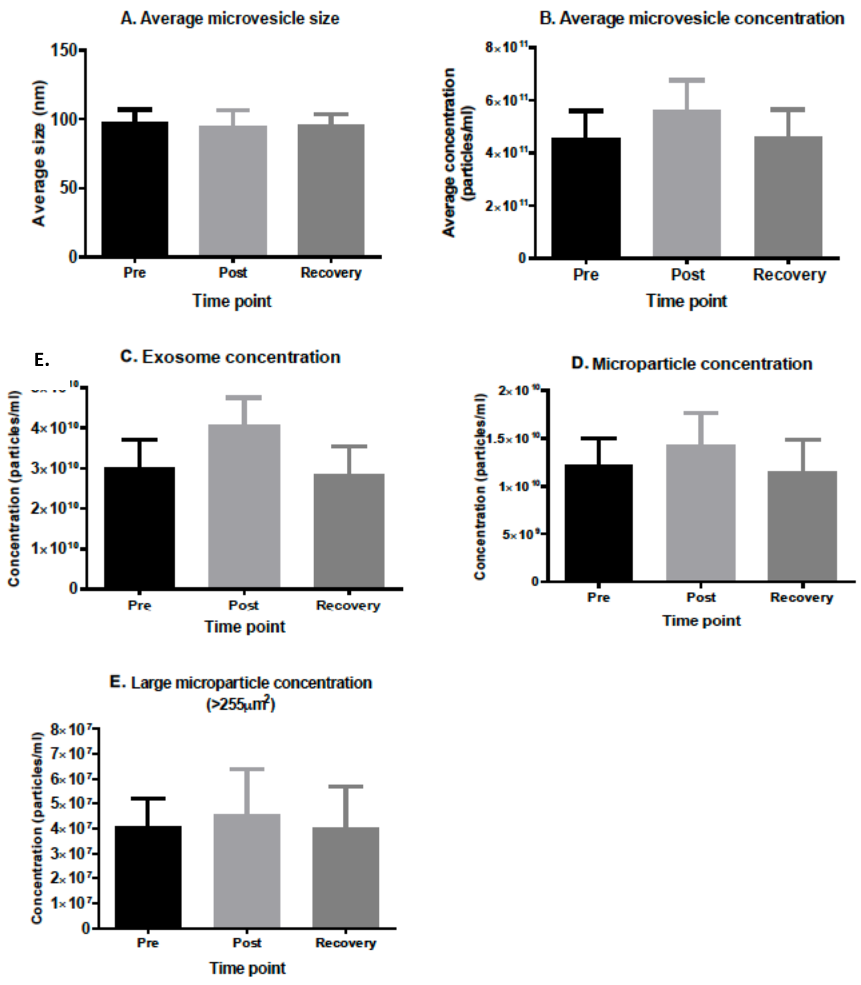

Figure S3: Effect of physical inactivity on microvesicle size and concentrations. Graphs represent the mean ± SEM of each parameter at each time point. A – Average microvesicle size, B – Average microvesicle concentration, C - Exosome concentration, D – Microparticle concentration and E – Large microparticle concentration. * P<0.05. Video capture (camera gain and shutter speed (1206), syringe pump speed (50)) and analysis (background subtraction, minimum track length) setting were adjusted, allowing optimal particle identification for platelet poor plasma. The number and duration of captures was set to 15 x 60 second captures, providing 15 replicates of the same sample. After the 15 videos for each sample were taken, the NTA software tracked the Brownian motion of individual vesicles by automatically locating and following the centre of each and every particle, measuring the average distance it moved per frame. NTA analysed the raw data, and calculated size and concentration and displayed different particle parameters (size versus relative intensity versus number) against each other. Settings were optimised and maintained between samples. For each 60 second video, the concentration and size of the particles (from 0-1000nm) were recorded. An experiment summary file was automatically generated, displaying the concentration of the sample at each vesicle size. The sum of the concentrations at each size were calculated and the average taken.

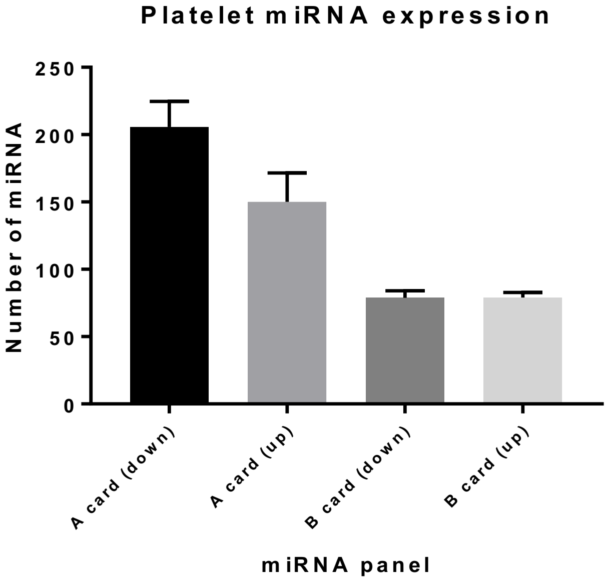

Figure S4: Quantification and confirmation of miRNA during the dry immersion study. Bar chart displaying the number of miRNA either up or downregulated ***END-***DI on the A and B panel, n = 8.

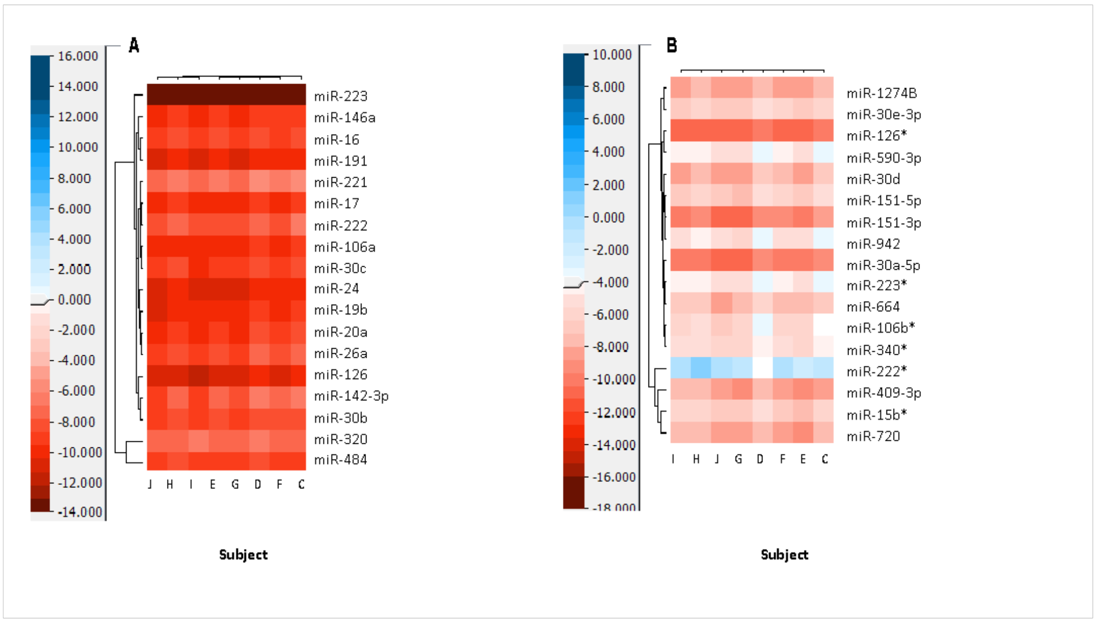

Figure S5: (A) Heat map showing the most highly abundant A panel platelet miRNA pre-DI. Each miRNA is listed on the righthand side and the subjects are listed on the bottom of the panel. The colour key is shown on the left. A change in colour from dark red to blue indicates a decrease in miRNA expression levels. **(B)** Heat map showing the most highly abundant B panel platelet miRNA before pre-DI.

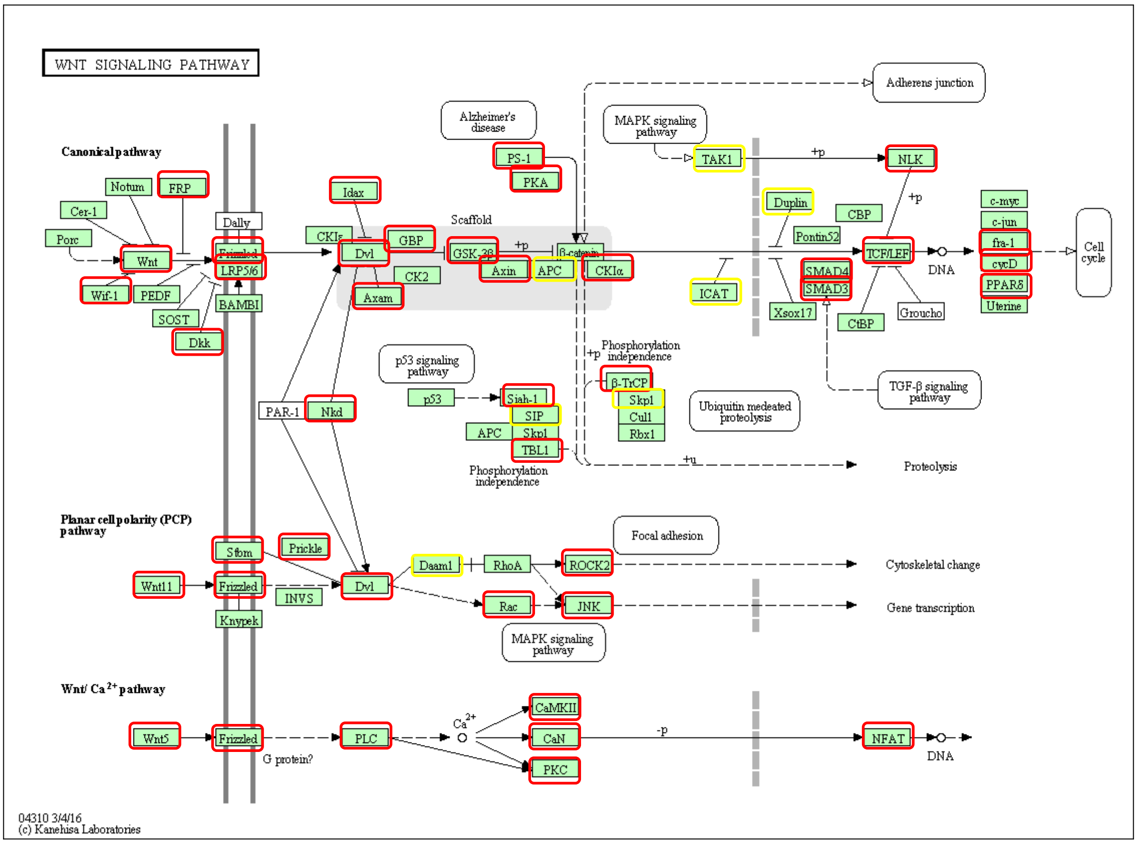

Figure S6: KEGG map of the Wnt Signalling pathway. Wnt signalling negatively regulates platelet function and miRNA targeting genes in this pathway were relevant to this study. Genes circled in red are predicted targets for multiple miRNAs, and genes circled in yellow are predicted targets of single miRNA. Permission was kindly granted by the Kanehisa Laboratory for the use of KEGG software.

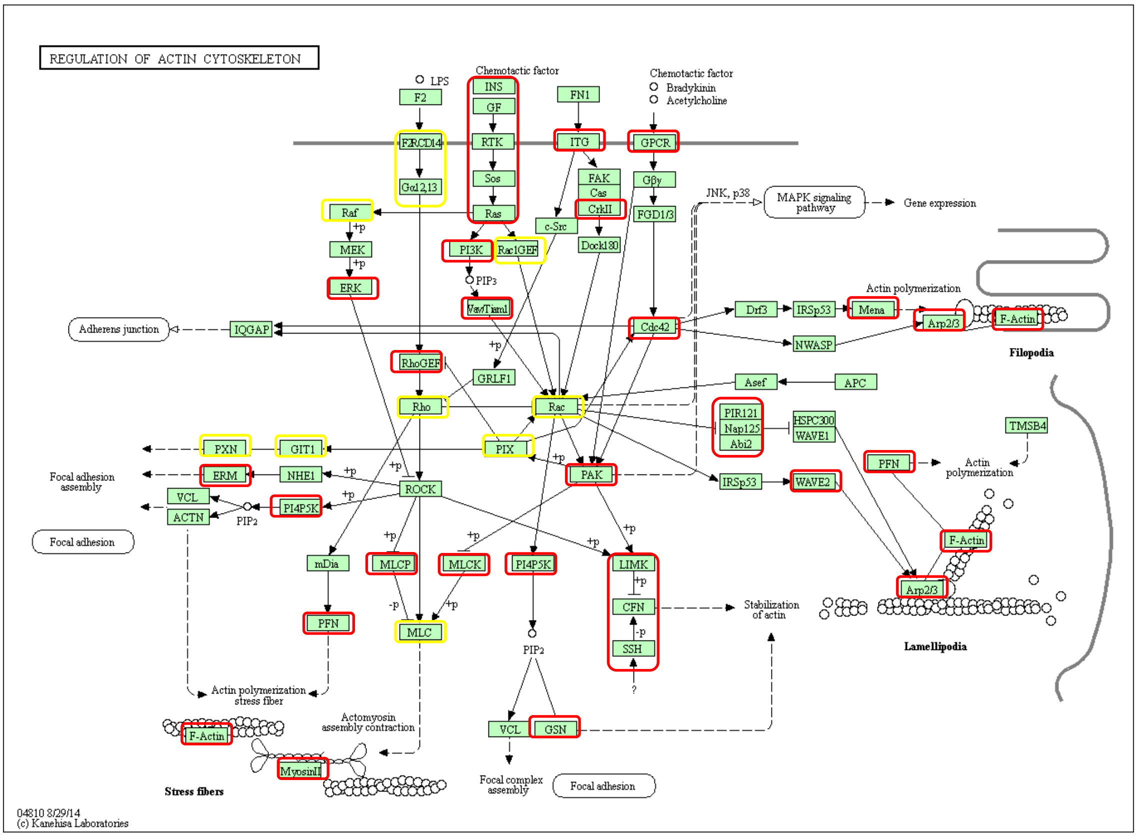

Figure S7: KEGG map of the regulation of actin cytoskeleton pathway. Reorganisation of the actin cytoskeleton is a key event in platelet activation and adhesion, and therefore this pathway was of interest with regard to platelet function. Genes circled in red are predicted targets for multiple miRNAs, and genes circled in yellow are predicted targets of single miRNA. Permission was kindly granted by the Kanehisa Laboratory for the use of KEGG software.

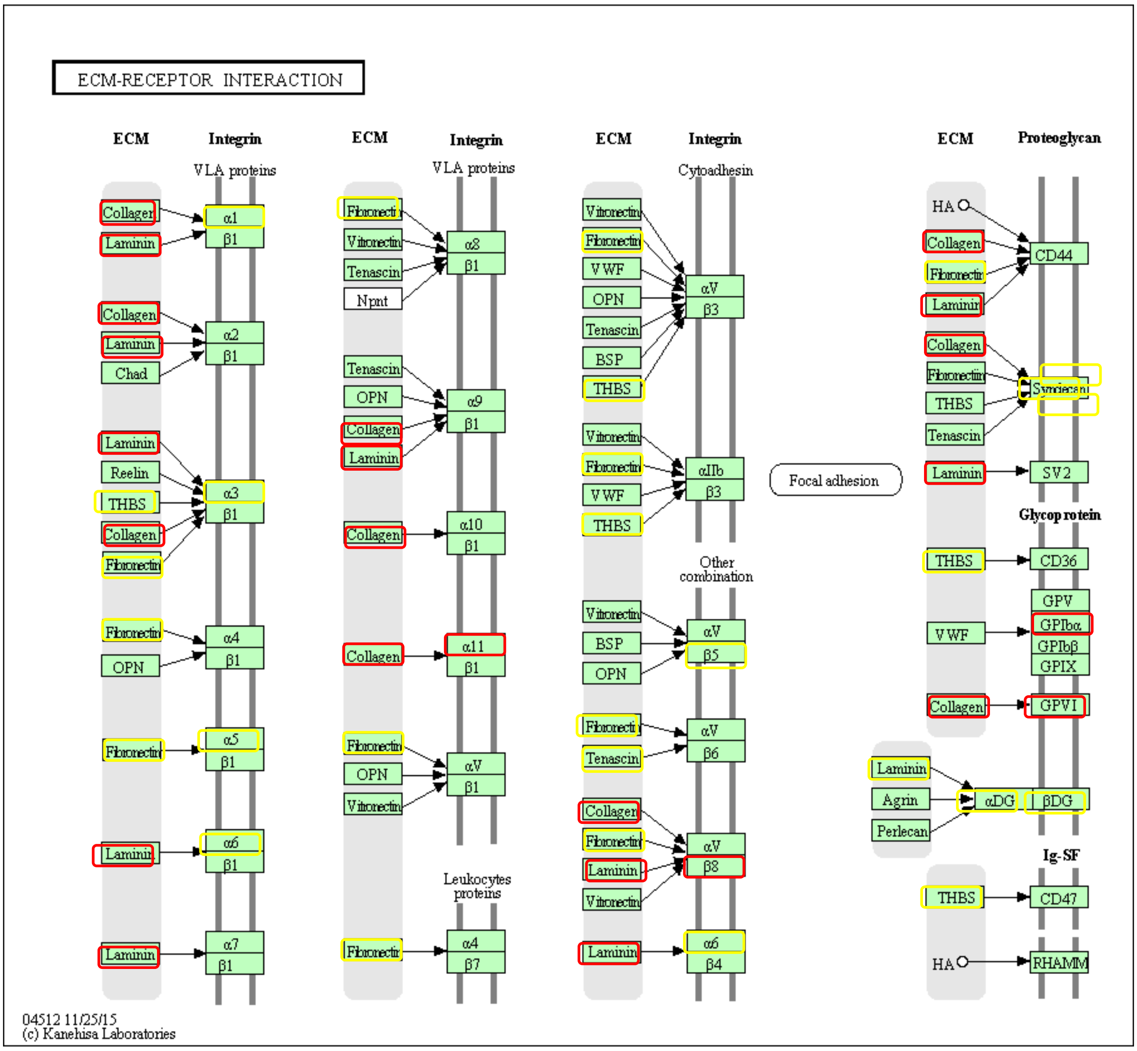

Figure S8: KEGG map of the ECM – receptor interaction pathway. Platelets express 5 different integrin's, which facilitate platelet adhesion to ECM proteins including collagen, laminin and fibronectin, amongst others, during platelet adhesion. This pathway was therefore chosen as it was relevant to platelet adhesion functions. Genes circled in red are predicted targets for multiple miRNAs, and genes circled in yellow are predicted targets of single miRNA. Permission was kindly granted by the Kanehisa Laboratory for the use of KEGG software.

SUPPLEMENTARY METHODS

Preparation of platelets

After blood draw, vacutainers/syringes were gently mixed by inversion. Blood was centrifuged w/o brake at 150xg for 10 minutes at RT. PRP was removed with a transfer pipette and placed into a fresh 50ml tube. Platelets were isolated from PRP by centrifugation, involving pelleting platelets and subsequent resuspension in a suitable buffer. The pH of PRP was brought to 6.5 using ACD. Prostaglandin E_1 (PGE_1), a platelet aggregation inhibitor, was added (1µl per 1 ml of PRP). PRP was centrifuged at 2000xg for 12 minutes with the brake on to pellet the platelets. The supernatant (PFP) was carefully removed and discarded. The platelet pellet was carefully resuspended in 1ml of buffer (either PBS/PFE/JNL) by gently pipetting up. A further 1ml of buffer was added with 2µl of PGE_1 and the washing step was repeated twice, adding PGE_1 before each wash. The platelet pellet was resuspended in buffer to the required concentration and platelets were allowed to sit at room temperature for 45 minutes to allow the PGE_1 to dissipate. Resuspended platelets were transferred to a fresh tube for further analysis.

Gel filtered platelets

For a very pure platelet population, gel filtered platelets were prepared. This is a gentler method of isolating platelets whereby they are passed through a gel filtration column of sepharose 2B [43].

Leukocyte (CD45) depletion of platelets

Washing platelets removes plasma contaminants, effectively diluting out plasma proteins and therefore leaving a pure platelet sample. Where a highly pure platelet population is required, further purification was necessary. For RNA analysis, the lesser RNA content of platelets may be contaminated by a relatively small number of white blood cells (WBCs). To prepare CD45-

depleted platelets for RNA analysis, EasySep™ magnetic technology was used. Cells are cross linked to EasySep™ magnetic particles using the Tetrameric Antibody Complex (TAC) technology and easily separated from unwanted message rich leukocytes (and CD45+ cells – a leukocyte specific marker) using the magnet. PFE Buffer (PBS + FBS + EDTA) was fresh each day RNA was prepared. For 19.5 ml of PFE buffer, 0.4 ml of FBS, 40 µl of 0.5 M EDTA and 19.5 ml of PBS were prepared in a 50 ml tube. This assay included the preparation of PRP as previously described. Platelets were suspended in 1 ml of PFE buffer after the second centrifugation step in a FACS tube. 1 ml of PFE buffer was added and mixed by gentle pipetting and platelets were counted. The EasySep™ whole blood depletion cocktail was then added to washed platelets. This sample was mixed gently and incubated for 15 minutes at RT. The EasySep™ magnetic nanoparticles were mixed vigorously to ensure they were in a uniform suspension and 200µl was added to the sample mixture. The sample was incubated at room temperature for 10 minutes. PFE buffer was added to a total volume of 5mls and the sample was mixed gently. The tube was placed into the magnet and incubated without the cap for 10 minutes at RT. The magnet was inverted, pouring off the supernatant into a 15ml RNAse-free tube. Platelets in the fresh tube were CD45-depleted platelets. 5 µl PGE_1 was added to the CD45-depleted platelets and mixed by inversion. The tube was centrifuged at 2000xg for 10 minutes at RT with the brake on to pellet the platelets. The plasma supernatant was removed, and the pellet was ready for RNA extraction.

Microvesicle quantification and analysis

The NanoSight NS300 and Syringe Pump were used to quantify microvesicles (exosomes and microparticles) in PFP samples. Nanoparticle tracking analysis technology (NTA) used in this device combines the properties of light scattering and Brownian motion to attain measurements including concentration and size distribution of particles in a liquid suspension. Particles in a beam path scatter light and are visualised by 20x magnification microscope fitted with a video camera. The camera functions at 30 frames per second and captures a video file

of the particles moving under Brownian motion. The software tracks particles individually and uses the Stokes-Einstein equation to calculate their hydrodynamic diameters and particle size-

$$Dt = \frac{K_B T}{6\pi n r_h}$$

Where Kb is Boltzmann's constant, T is temperature, n is solvent viscosity, and Dt is particle diffusion coefficient (hence sphere equivalent). The syringe was rinsed with 1ml of 10 % EtoH followed with 1ml of PBS. This procedure was performed between each sample. A 1:2500 dilution of PFP and high pure PBS was used after serial dilution optimisation (dilution 1=4µl of sample and 996µl of PBS, dilution 2=100µl of dilution 1 and 900µl of high pure PBS). Samples were kept on ice until analysis.
